# Comparative metabolomics of high-unsaturated fatty acid oils: exploring volatile and nonvolatile metabolite differences in olive, walnut, flaxseed, and camellia oils

**DOI:** 10.3389/fnut.2026.1853929

**Published:** 2026-08-03

**Authors:** Bo Zhang, Zhanglian Chen, Kai Qin, Yongxing Yang, Li Xu, Zhengwu Zhang

**Affiliations:** 1 Longnan Academy of Non-wood Forest, Longnan, China; 2 Key Laboratory of Agricultural Product Processing and Quality Control of Specialty (Co-construction by Ministry and Province), School of Food Science and Technology, Shihezi University, Shihezi, Xinjiang, China

**Keywords:** camellia oil, flavor profile, flaxseed oil, high-unsaturated fatty acid oils, metabolomics, olive oil, walnut oil

## Abstract

**Introduction:**

Olive, walnut, flaxseed, and camellia oils are premium edible oils rich in unsaturated fatty acids, yet they exhibit distinct fatty acid profiles: olive and camellia oils are high in monounsaturated fatty acids, while walnut and flaxseed oils are abundant in polyunsaturated fatty acids. Despite their market value and nutritional importance, a systematic comparative metabolomic analysis covering both volatile and non‑volatile compounds under unified conditions has been lacking.

**Methods:**

We performed a comprehensive metabolomic comparison of the four oils using headspace solid‑phase microextraction/gas chromatography‑mass spectrometry (HS‑SPME/GC‑MS) for volatile compounds and ultra‑high‑performance liquid chromatography‑high‑resolution mass spectrometry (UHPLC‑HRMS) for non‑volatile metabolites. Multivariate statistical methods, including principal component analysis (PCA), partial least‑squares discriminant analysis (PLS‑DA), and relative odor activity value (ROAV) calculation, were applied to identify differential metabolites and characteristic biomarkers.

**Results:**

A total of 100 volatile and 1,186 non‑volatile metabolites were identified, covering alcohols, aldehydes, fatty acids, terpenes, phenolics, and others. PCA and heatmap analyses showed clear separation among the four oils. Common flavor markers included hexanal, ethanol, and ethyl hexanoate, while specific biomarkers such as hydroxytyrosol (olive oil), juglone (walnut oil), lignans (flaxseed oil), and camelliasaponin (camellia oil) were distinguished. ROAV analysis highlighted key flavor‑active volatiles, and differential metabolites were significantly correlated with flavor attributes, oxidative stability, and nutritional characteristics.

**Discussion:**

Our integrated volatile‑non‑volatile metabolomic approach provides a systematic foundation for comparing the compositional, flavor, and quality differences among high‑unsaturated fatty acid vegetable oils. The identified biomarkers and their correlations offer valuable tools for oil authentication, quality control, and functional product development, demonstrating the superiority of combined metabolomic profiling over single‑platform analyses.

## Graphical abstract

**Figure:**
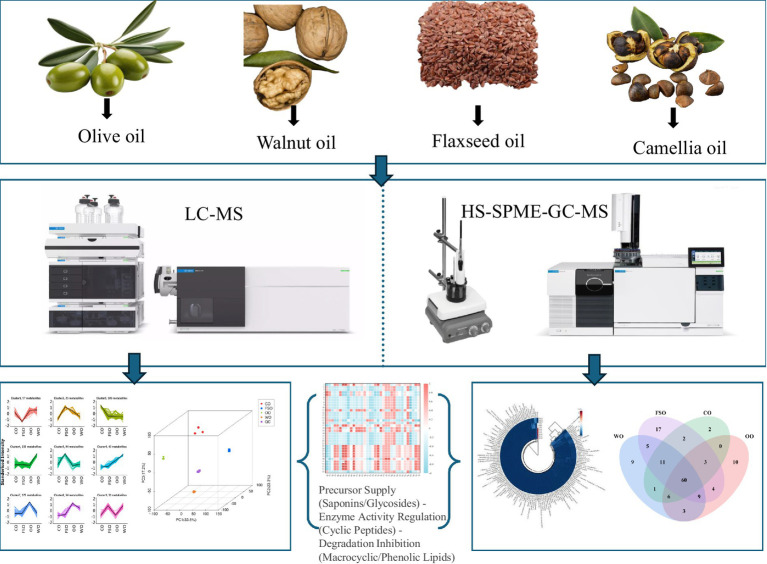

## Highlights

- HS-SPME/GC–MS and UPLC–HRMS reveal 100 volatile and 1186 non-volatile metabolites in four vegetable oils.
- Multivariate analysis distinguished oil profiles by volatile content, fatty acid composition, and key metabolites.
- ROAV ≥1 compounds: 9 (WO), 9 (OO), 15 (FSO), 6 (CO); ethanol, 1-octen-3-ol, hexanal etc. shared as key aroma components.
- Characteristic non-volatiles: hydroxytyrosol (OO), juglone (WO), lignans (FSO), camelliasaponin (CO), linked to nutrition/stability.
- Pearson analysis showed cross-regulation: glycosides supply lipid precursors, cyclic peptides regulate enzymes, alkaloids activate oxidase.

## 1 Introduction

Edible oils play a crucial role in nutrition and health, serving as a primary source of fats and energy in the human diet, as well as carriers of essential fatty acids and fat-soluble vitamins. Their nutritional profiles can significantly influence health outcomes by aiding in the prevention and management of various diseases. Extra virgin olive oil is particularly renowned for its health benefits, largely attributed to its high content of monounsaturated fats, especially oleic acid, which is known to promote heart health. Additionally, olive oil is rich in antioxidants, such as vitamin E and polyphenols, which have demonstrated anti-inflammatory properties that help combat oxidative stress (1). Walnut oil is rich in linoleic acid (50–65%) and alpha-linolenic acid (9–14%), with an *ω*-6/ω-3 ratio ranging from 4:1 to 6:1, which is close to the ideal intake ratio for humans (2). In contrast to the high oleic acid and low *ω*-3 characteristics of olive oil, walnut oil also contains abundant bioactive compounds such as gamma-tocopherol, polyphenols, phytosterols, and squalene, which bestow it with stronger antioxidant, anti-inflammatory, and intestinal barrier protection functions (3). Flaxseed oil is particularly valued for its high omega-3 fatty acid content, making it an important source of ALA. This oil is beneficial in reducing the risk of cardiovascular diseases and promoting overall wellness. Moreover, it contains lignans, which possess antioxidant properties and may contribute to reducing the risk of hormone-related cancers (4). Camellia oil, extracted from the seeds of the Camellia oleifera plant, is rich in oleic acid (approximately 80% or more), similar to olive oil, and has garnered attention for its potential health benefits. It contains bioactive compounds such as squalene, tocopherols, and polyphenols, which offer antioxidant and anti-inflammatory properties. In summary, the health benefits of these oils stem from their unique compositions of fatty acids, antioxidants, and bioactive compounds. The four oil matrices were deliberately selected for validating this integrated metabolomics strategy based on distinct compositional gradients. Olive and camellia oils are oleic acid-enriched matrices, whereas walnut and flaxseed oils are dominated by linoleic acid and *α*-linolenic acid, creating two contrasting fatty acid profiles to distinguish lipid-derived metabolites. Moreover, each oil contains exclusive signature bioactive compounds: hydroxytyrosol in olive oil, juglone in walnut oil, lignans in flaxseed oil, and camelliasaponin in camellia oil. Their volatile flavor profiles also differ drastically in alcohols, aldehydes and terpenoids. This dual gradient of fatty acid composition and unique matrix-specific metabolites provides an ideal comparative system to verify the reliability and superiority of combined volatile and non-volatile metabolomics over single profiling analysis.

Research has extensively characterized the quality, flavor, and functional attributes of individual polyunsaturated fatty acid (PUFA)-rich vegetable oils. However, most studies have focused on individual oil varieties and separately analyzed either volatile or non-volatile compounds. Systematic comparative research combining volatile and non-volatile metabolomics under unified experimental conditions is still lacking. This limitation prevents a full understanding of the intrinsic links between oil flavor, nutrition and stability. The core competitiveness of vegetable oils rich in unsaturated fatty acids is attributed not only to the nutritional benefits conferred by polyunsaturated fatty acids (PUFAs), such as their role in regulating blood lipid levels and their anti-inflammatory properties, but also to the unique sensory experiences they offer, exemplified by the grassy aroma of flaxseed oil (5). Beyond polyunsaturated fatty acids (PUFAs), vegetable oils also contain non-volatile active components, including phenolic compounds (e.g., lignans found in flaxseed), sterols, and vitamin E. These substances not only inhibit PUFA oxidation but also synergistically enhance nutritional effects. Analyzing this non-volatile metabolic profile elucidates the types and distribution of these active components, thereby directly providing data that supports the nutritional value of the oils. In vegetable oils rich in unsaturated fatty acids, the co-presence and interrelated profiles of volatile and non-volatile metabolites form a critical basis for linking sensory quality, oxidative stability, nutritional function, and safety.

Metabolomic approaches targeting volatile and non-volatile components are powerful tools for studying edible oils, facilitating quality assessment (6), variety and origin tracing, oxidation stability evaluation (7), nutritional and functional analysis (8), and optimization of processing and storage (9). To address the aforementioned research gap, this study puts forward an explicit scientific hypothesis: conventional fatty acid profiling only characterizes fundamental lipid compositions and is incapable of capturing holistic metabolic fingerprints governing the sensory attributes, antioxidant activity and nutritional potency of edible oils. By contrast, the combinatorial profiling of volatile and non-volatile metabolites enables the screening of matrix-exclusive diagnostic biomarkers and the elucidation of interrelated metabolic regulatory pathways undetectable via mono-layer analytical strategies, which represents the primary innovation of the present work.

This study investigates four types of vegetable oils that are rich in highly unsaturated fatty acids: olive oil, walnut oil, flaxseed oil, and camellia oil. Utilizing metabolomics technology, which focuses on the identification of metabolites and their functions within biological systems, this research employs a core analytical approach. By conducting a comparative analysis of both volatile and non-volatile metabolites, the study aims to clarify the chemical basis for the differences in health effects among these oils. A thorough comparative analysis is performed on vegetable oils with high unsaturated fatty acid content, emphasizing the screening of characteristic metabolites that are directly related to, or significantly differ in, the health benefits and potential risks associated with these oils.

## 2 Materials and methods

### 2.1 Materials and reagents

Walnut kernels (variety: ‘Longnan No.15’), flaxseeds (variety: ‘Dingya No.26’), and ripe olives (variety: ‘Leccino’) were sourced from the agricultural market in Longnan, Gansu Province. These samples were cultivated in a semi-arid continental climate and were harvested in September 2024. Camellia seeds (variety: ‘Hunan Hongye’) were obtained from Tianwo Technology Co., Ltd. in Hunan, where they were grown in a subtropical monsoon climate, and were harvested in November 2024. To guarantee sample representativeness and experimental reproducibility, three independent replicate batches for each raw material were additionally collected from different commercial suppliers. Walnut kernels and flaxseeds were obtained from the Tianshui Agricultural Wholesale Market (Gansu), ripe olives from the Lanzhou Imported Agricultural Products Market (Gansu), and camellia seeds from Hunan Lvfeng Agricultural Development Co., Ltd. (Hunan). All supplementary samples shared the same cultivar, growth environment and harvest season as the original materials. To eliminate metabolite variations induced by inconsistent post-harvest storage of market-sourced and factory-supplied raw materials, unified cold-chain and storage specifications were applied to all batches. All raw materials were transported under sealed low-permeability packaging at 10 °C cold chain within 72 h post-harvest. After delivery to the laboratory, all kernels/seeds were vacuum-sealed in light-proof aluminum foil bags and stored at 4 °C, with the storage period strictly limited to ≤7 days before pretreatment. No raw material was exposed to ambient air for more than 30 min during sorting and cleaning to prevent spontaneous oxidation and volatile loss. Identical crushing, drying, pressing and preservation parameters were adopted across all replicate batches to exclude handling differences as confounding factors.

Walnut kernels, flaxseeds and camellia seeds were rinsed once with distilled water. Subsequently, the samples were dried in a vacuum drying oven (Model DZF-6020, Xiniu Technology Co., Ltd., Shanghai, China) at 40 °C for 1 h to remove surface moisture. The low-temperature drying treatment at 40 °C for 1 h efficiently eliminated superficial moisture, and this mild pretreatment exerted negligible adverse effects on endogenous volatile compounds, which was fully compatible with subsequent volatile metabolomics detection. Fresh ripe olives were only gently blotted with filter paper to remove free surface water without vacuum drying, consistent with conventional cold-pressing techniques for olive oil; multi-stage centrifugation and phase separation during extraction further removed excess water, and the moisture content of final olive oil was steadily below 0.2%, meeting the quality standard for extra virgin olive oil.

The processed walnut, flax and camellia raw materials were then crushed into small particles of 2–3 mm using a high-speed grinder (Model FTT-2500G, Dongguan Fangtai Electric Co., Ltd., China). A screw oil press (Model ZYJ-420, Dongguan Fangtai Electric Co., Ltd., China) was used for cold pressing with the temperature set to 30 °C. For each batch, 2.0 kg of crushed raw materials were fed in batches to extract crude oil. The obtained crude oil was transferred to centrifuge tubes and centrifuged at 8000 rpm for 15 min to separate the oil phase. The upper oil phase was collected and dehydrated with 3% anhydrous sodium sulfate. After uniform shaking, the mixture was centrifuged at 4000 rpm for 5 min to acquire clarified oil samples.

The olive oil extraction procedure described herein was modified according to (10). A total of 750 g of fresh olive fruits were crushed into pulp using a grinding mill. The obtained pulp was placed in a water bath system and continuously stirred at 28 °C for 60 min. Thereafter, the pulp was centrifuged twice at 3500 rpm for 1 min per cycle. The centrifuged liquid was collected in a graduated cylinder and stood still for natural phase separation. The upper oil phase was harvested and stored in brown reagent bottles at −20 °C for standby use.

All clarified oils were promptly transferred into brown reagent bottles and tightly sealed at corresponding storage temperatures. Prior to sealing, each oil sample was purged with nitrogen to isolate light and oxygen, which effectively suppressed lipid oxidation and the degradation of endogenous metabolites. All metabolomic measurements were conducted within 7 days under constant low-temperature preservation to sustain the stability of both volatile and non-volatile metabolites during the entire experiment.

Methanol, acetonitrile, and isopropanol were all of LC–MS grade and purchased from CNW Technologies. Acetic acid, also of LC–MS grade, was sourced from SIGMA-ALDRICH. Ultrapure water was obtained from Watsons.

### 2.2 GC–MS analysis and odor activity value analysis

Volatile compounds were extracted using the headspace solid-phase microextraction (HS-SPME) technique. The specific procedure is as follows: 1000 μL of the oil sample was added to a 20 mL headspace sample vial, followed by the addition of 15 μL of an internal standard solution of 2-octanol (TCI, 10 mg/mL stock solution dissolved in H_2_O) for semi-quantitative analysis. Due to the high complexity of volatile components in oil samples, it is infeasible to obtain pure reference standards for every detected compound. Subsequently, the SPME fiber (DVB/CAR/PDMS, 50/30 μm, Supelco Inc., Bellefonte, PA, USA) was inserted into the headspace container to adsorb the volatile compounds. The extraction temperature was set at 60 °C, with preheating and extraction times of 15 min and 30 min, respectively. Finally, the fiber was inserted into the GC–MS injector for a 4-min thermal desorption. The HS-SPME extraction conditions were adopted from previously reported methods (11).

The volatile metabolites of four vegetable oil samples were analyzed using the Agilent 7,890 B-5977 B GC–MS system (Agilent Technologies, Santa Clara, CA, USA), which was equipped with a DB-WAX column (30 m length, 0.25 mm internal diameter, and 0.25 μm film thickness). The analysis was conducted in splitless mode, with the injector temperature maintained at 250 °C and a septum purge flow rate of 3 mL/min. The initial oven temperature was set at 40 °C and held for 4 min, followed by an increase to 245 °C at a rate of 5 °C/min, which was then held for an additional 5 min. The temperatures of the transfer line and ion source were adjusted to 250 °C and 230 °C, respectively, while the quadrupole temperature was set to 150 °C. The mass range was m/z: 20–400, the scan mode was set to ‘Scan’, and the solvent delay was configured to 2.37 min.

A mixture of n-alkane standards (10 μL, 50 mg/L) was used to calculate retention indices (RI, Supplementary Table S1). Volatile compounds were reliably identified via combined matching of retention indices (RI), characteristic fragment ions and full mass spectra against the NIST standard database (Gaithersburg, MD, USA). The matching threshold for spectral similarity was set above 80%. The mass spectrometry data were analyzed for peak extraction, baseline correction, deconvolution, peak integration, peak alignment, and mass spectrum matching using Chroma TOF software (V 4.3x, LECO) and the NIST library. The relative concentration of each metabolite was calculated using total ion current (TIC) peak area normalization. Specifically, the peak area of each individual metabolite was divided by the total peak area of all detected metabolites within the corresponding sample, resulting in a ratio defined as the relative concentration. The relative concentrations of volatile metabolites and their odor activity values (ROAV) are calculated using the following formulas from (12):


$$\text{ROAV}=100\times \frac{C{\%}_{A}}{C{\%}_{\text{stan}}}\times \frac{T_{\text{stan}}}{T_{A}}$$

C%_A_: The relative percentage content of the average peak area of other compounds. C%_stan_: The relative percentage content of the average peak area of the compound that contributes the most to the overall flavor of the sample group. T_stan_: The sensory threshold of the compound that contributes the most to the overall flavor of the sample group. T_A_: The sensory threshold of other compounds. The ROAV of all compounds are ≤100, indicating that a higher ROAV value signifies a greater contribution of the compound to the overall flavor of the sample. Literature generally considers compounds with ROAV ≥ 1 to be the key flavor compounds in the analyzed sample group, while those with 0.1 ≤ ROAV < 1 are regarded as playing a significant modifying role in the overall flavor of the sample group.

### 2.3 UHPLC-Q-Exactive/MS-based metabolomics analysis

#### 2.3.1 Sample preparation

Transfer 100 μL of the liquid sample into an EP tube, and add 400 μL of extraction solution (methanol:acetonitrile = 1:1, v/v), which contains isotopically labeled internal standards. The methanol-acetonitrile solvent system was selected to target polar and medium-polarity metabolites in oil samples, including phenolic compounds, tocopherols, phytosterols and polar lipid derivatives. The mixture was vortexed vigorously for 30 s to achieve thorough blending, followed by ultrasonic extraction in an ice-water bath for 10 min to avoid metabolite degradation. Afterwards, the sample was kept static at −40 °C for 1 h to fully precipitate proteins and macromolecular impurities. After standing, 350 μL of the supernatant was transferred into the wells of a protein precipitation plate. The protein precipitation plate was tightly assembled with a collection plate and placed into a positive pressure processing device. The pressure was set to 6 psi, and positive pressure filtration was performed for 120 s. The resulting filtrate was collected and immediately stored at 4 °C pending UHPLC–MS analysis. The metabolite extraction method was based on published protocols (13).

#### 2.3.2 UPLC–MS/MS

The liquid chromatography-mass spectrometry (LC–MS) analysis was conducted using a Vanquish-Orbitrap Exploris 120 ultra-high performance liquid chromatography mass spectrometer (Thermo Fisher Scientific, Waltham, MA, USA), which was equipped with a Phenomenex Kinetex C18 column (2.1 mm × 50 mm, 2.6 μm particle size; Phenomenex Inc., Torrance, CA, USA). The autosampler temperature was maintained at 4 °C, while the column temperature was set to 25 °C. The flow rate and injection volume were established at 0.3 mL/min and 2 μL, respectively. For the LC-ESI-MS analysis, mobile phase A consisted of 0.01% acetic acid in water, and mobile phase B was a mixture of isopropanol and acetonitrile (1:1, v/v). The separation gradient was as follows: 0–0.5 min, 1% B; 0.5–4 min, 1–99% B; 4–4.5 min, 99% B; 4.5–4.55 min, 99–1% B; and 4.55–6 min, 1% B. The Orbitrap Exploris 120 mass spectrometer was operated using Xcalibur control software (version 4.4, Thermo) for the acquisition of both primary and secondary mass spectrometry data, with the following specific parameters: sheath gas flow rate of 50 Arb, auxiliary gas flow rate of 15 Arb, capillary temperature of 320 °C, full MS resolution of 60,000, MS/MS resolution of 15,000, collision energy set at SNCE 20/30/40, and spray voltage of 3.8 kV (positive) or −3.4 kV (negative).

#### 2.3.3 Data processing

The raw data were initially converted to mzXML format using the ProteoWizard software package (v3.0.8789). Subsequently, the R XCMS software package was employed for feature detection, retention time correction, and alignment. Metabolites were identified based on accurate mass numbers (ppm < 30 ppm) and MS/MS data, subsequently matched with databases including HMDB^1^, MassBank^2^, LipidMaps^3^, and mzCloud^4^. Robust LOESS signal correction (QC-RLSC) was utilized for data normalization, addressing any systematic biases. Following normalization, only ion peaks with a relative standard deviation (RSD) of less than 30% in quality control (QC) were retained to ensure accurate metabolite identification. All samples and QC samples were analyzed in random injection order throughout the LC–MS run to eliminate instrument drift.

### 2.4 Statistical analysis

Analysis of variance (ANOVA) was conducted using SPSS 26.0 (IBM Corporation, Armonk, NY, USA). Principal component analysis (PCA) and partial least squares-discriminant analysis (PLS-DA) were performed using SIMCA-14.1 software (Umetrics AB, Sweden). The PLS-DA model was validated by seven-fold cross-validation and 200 permutation tests to avoid overfitting and ensure model reliability. PCA was utilized to elucidate the differences among various batches of vegetable oils, while the PLS-DA model was employed to calculate the variable importance in projection (VIP) values. Compounds with VIP values greater than 1.0 and *p* < 0.05 were classified as significantly differential metabolites (SDMs). This dual-criterion screening strategy (VIP and *p*-value) is widely accepted in metabolomics research to screen differential markers comprehensively and credibly. Finally, Origin 2021 (Northampton, Massachusetts, USA) and OmicShare online tools^5^ were utilized for data visualization.

## 3 Result and discussion

### 3.1 Identification and comparison of volatile compounds

#### 3.1.1 Volatile compound analysis by GC–MS

Supplementary Figure S1 presents the representative total ion current (TIC) chromatograms of volatile compounds in four vegetable oils (camellia oil, flaxseed oil, olive oil, and walnut oil) analyzed by HS-SPME-GC–MS. All samples showed well-separated chromatographic peaks, and distinct volatile fingerprint differences were observed among the four oil samples.

**Figure 1:**
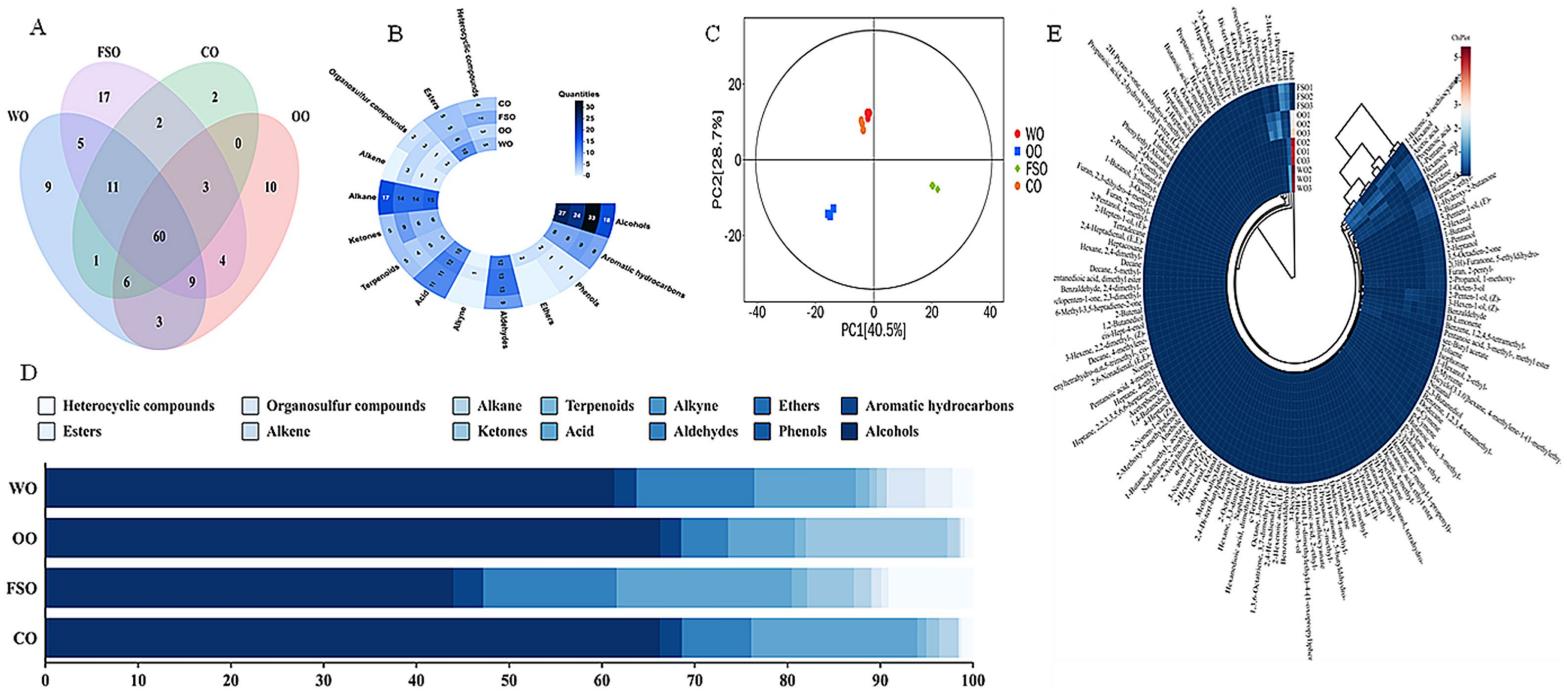
Comparative analysis of volatile compounds in four vegetables oils: walnut oil (WO), olive oil (OO), flaxseed oil (FSO), and camellia oil (CO). **(A)** Venn diagram illustrating the number of unique and shared volatile compounds among the four oils; **(B)** Principal component analysis (PCA) score plot based on volatile profiles. **(C)** Histogram showing the total number of volatile compounds categorized by chemical classes in each oil; **(D)** Stacked bar chart representing the relative abundance of volatile compound classes across the four oils; **(E)** Heatmap combined with hierarchical clustering of volatile compounds.

The volatile components of four edible oils were analyzed using headspace solid-phase microextraction coupled with gas chromatography–mass spectrometry (HS-SPME/GC–MS), as shown in Figure 1. A total of 100 volatile components were identified in walnut oil, comprising 27 alcohols, 15 alkanes, 12 aldehydes, 10 esters, 10 acids, 6 ketones, and 20 other compounds, consistent with the findings reported by Sun et al. (47). In olive oil, 88 volatile components were detected, including 24 alcohols, 13 alkanes, 13 aldehydes, 6 esters, 12 acids, 6 ketones, and 15 others. This composition is consistent with established literature on extra virgin olive oil (EVOO), where C6-C9 aldehydes such as hexanal, (E)-2-hexenal, and (E, E)-2,4-decadienal, along with alcohols like 1-hexanol and trans-2-hexen-1-ol, and ketones such as 1-octen-3-one, are known to contribute to its characteristic “green” and “fruity” aroma families (14). Flaxseed oil contained 107 volatile components, with 33 alcohols, 13 alkanes, 13 aldehydes, 5 esters, 11 acids, 9 ketones, and 23 others. The abundance of terpenes like *β*-myrcene and branched aldehydes in flaxseed oil is consistent with recent metabolomic profiling studies (15). Hexanal, with a concentration of 0.662 mg/mL, was also significant in flaxseed oil (15). Camellia oil exhibited 79 volatile components, comprising 18 alcohols, 16 alkanes, 9 aldehydes, 5 esters, 11 acids, 5 ketones, and 15 others. Figure 1A illustrates the shared and unique components of volatile compounds among the four oils (Walnut Oil (WO), Olive Oil (OO), Flaxseed Oil (FSO), and Camellia Oil (CO)). The Venn diagram indicates that there are 2 shared compounds between FSO and CO, 2 shared compounds between WO and FSO, 3 shared compounds between WO and CO, and the highest number of shared compounds, 10, between WO and OO. Additionally, each oil possesses a unique number of compounds, with WO having the most unique compounds at 60, followed by FSO (11), CO (9), and OO (5). Figure 1B presents a petal diagram that elaborates on the distribution of various categories of volatile compounds across the four oils. The figure effectively illustrates the quantities of alcohols, alkanes, aldehydes, esters, acids, ketones, phenols, and other compounds present in these oils. It is evident that alcohols, alkanes, and aldehydes are the predominant categories of volatile compounds in all oils, whereas the distribution of ketones and phenols is comparatively lower. Figure 1D depicts the total amounts of different categories of volatile compounds in the four edible oils. The figure indicates that alcohols and alkanes are the most significant categories of volatile compounds across all oils, while the total amounts of ketones, phenols, and esters are relatively lower. WO exhibits the highest total amounts of alcohols and alkanes, whereas FSO shows comparatively elevated total amounts of acids and esters.

Supplementary Table S4 provides detailed information on the retention indices (RI), molecular formulas, CAS numbers, concentrations (mg/mL), thresholds (ppb), and relative aroma contribution values (ROAV) of each volatile component. The primary source of alcohols is the decomposition of fatty acids, which are significant products of the oxidation process of vegetable oils, imparting them with distinct plant and aromatic scents (16). Ethanol is found in the highest concentration in walnut oil (5.297 mg/mL), while its levels are lower in other oils. The concentration of 1-hexanol in walnut oil is 0.581 mg/mL, whereas it is undetectable in olive oil and camellia oil. The aldehydes present in oils arise from the transformation of their high unsaturated fatty acid content into hydroperoxides via lipoxygenase (LOX), which are subsequently degraded into aldehydes under the catalytic conditions of hydroperoxide lyase (HPL). Hexanal is relatively abundant across all four oils, with walnut oil containing 1.085 mg/mL, olive oil 0.194 mg/mL, flaxseed oil 0.662 mg/mL, and camellia oil 0.414 mg/mL. Furthermore, aldehydes possess low odor thresholds; for instance, hexanal has a threshold of 4 ppb, which significantly contributes to the oil’s flavor profile. Ester compounds primarily provide fruity and floral aromas to oils, predominantly resulting from the esterification reaction between alcohols and acids (48). The concentration of ethyl acetate in walnut oil is 0.133 mg/mL, while it remains undetected in other oils. The level of ethyl hexanoate in flaxseed oil is 0.011 mg/mL, which is lower than that in other oils.

Ketone compounds primarily arise from fat oxidation and the Maillard reaction; however, their thresholds are significantly higher than those of their isomeric aldehydes, contributing minimally to the overall flavor profile. The concentration of 3-pentanone in olive oil is measured at 0.464 mg/mL, whereas it remains undetected in other oils. In contrast, the concentration of 2-heptanone in flaxseed oil is 0.041 mg/mL, which is lower than that found in other oils. Data analysis reveals that the levels of certain volatile metabolites differ markedly among various oils, and these metabolites may serve as potential quality markers. For instance, the ethanol concentration in walnut oil is considerably higher than in other oils, suggesting its potential as a marker for walnut oil. Additionally, hexanal, which contributes a fruity aroma complemented by green, leafy, and woody notes, is present in the highest concentration in walnut oil, thereby serving as another potential marker. Furthermore, ethyl acetate is exclusively detected in walnut oil, reinforcing its status as a potential marker for this oil.

By calculating the relative aroma contribution value (ROAV) of each volatile component, its contribution to the oil flavor is assessed. In walnut oil, there are 9 substances with ROAV > 1, which are Ethanol, 1-Hexanol, 1-Octen-3-ol, Hexanal, Nonanal, Pentanal, Benzaldehyde, 2,6-Nonadienal, (E, E)-, and Hexanoic acid, ethyl ester. In olive oil, there are 9 substances with ROAV > 1, which are Ethanol, 1-Hexanol, 1-Octen-3-ol, 1-Heptanol, Hexanal, Nonanal, Benzaldehyde, Butanal, 2-methyl-, 1-Penten-3-one, and Hexanoic acid, ethyl ester. In flaxseed oil, there are 15 substances with ROAV > 1, which are 1-Pentanol, 1-Octen-3-ol, 1-Penten-3-ol, Benzyl alcohol, Hexanal, Nonanal, Pentanal, Benzaldehyde, Butanal, 2-methyl-, 2-Pentenal, (E)-, 2,6-Nonadienal, (E, E)-, Hexanoic acid, *β*-Myrcene, 2-Heptanone, 1-Penten-3-one, Furan, 2-pentyl-, and Hexanoic acid, ethyl ester. In camellia oil, there are 6 substances with ROAV> 1, which are Ethanol, 1-Octen-3-ol, Hexanal, Nonanal, Pentanal, and Hexanoic acid, ethyl ester. Ethanol, 1-Octen-3-ol, Hexanal, Nonanal, and Ethyl hexanoate are the characteristic aroma components shared by the four vegetable oils. Among them, Hexanal, which has a green, herbal scent, exhibits high ROAV values in all four oils, indicating its significant contribution to the flavor of the oils. Ethanol shows a high ROAV value in walnut oil, while its contribution is minimal in the other oils. Propionic acid has low ROAV values across all four oils, suggesting its minor role in flavor contribution. The characteristic substances of walnut oil are 1-Hexanol, which has a slight floral aroma, and Benzaldehyde, which has an almond scent. The characteristic substances of olive oil are 1-Heptanol, which has a mild fruity aroma, and Butanal, 2-methyl-, which has a slight sweet fragrance. The characteristic substances of flaxseed oil are 1-Pentanol, which has a mild fruity aroma, and *β*-Myrcene, which has a fresh pinewood scent. The differences in the types of volatile components and their ROAV values in different oils reflect their unique flavor profiles. These volatile components not only influence the aroma and taste of the oil but may also affect its nutritional value and stability. Future research could further explore the specific mechanisms of action of these volatile components and how to improve the overall quality of edible oil by regulating their composition.

#### 3.1.2 Analysis of volatile compound differences

Figure 1C presents the principal component analysis (PCA) score plot for four edible oil samples. The plot illustrates the first two principal components, PC1 and PC2, which account for 40.5 and 28.7% of the total variance, respectively. The distinct separation of the different oil samples on the score plot indicates significant differences in their volatile compound compositions. Specifically, WO and OO exhibit higher scores on PC1, whereas FSO and CO show higher scores on PC2, further confirming their chemical composition differences. Figure 1E displays a heat map representing the relative contents of 80 volatile compounds across the four edible oils. The color intensity in the heat map reflects the compound content, with red indicating high content and blue indicating low content. Notably, significant differences in the distribution of compound contents among the various oils are observed, consistent with the PCA analysis results. Additionally, the heat map reveals that certain specific compounds are present in high concentrations in particular oils, which may be attributed to the oil processing techniques or the characteristics of raw materials.

**Figure 2:**
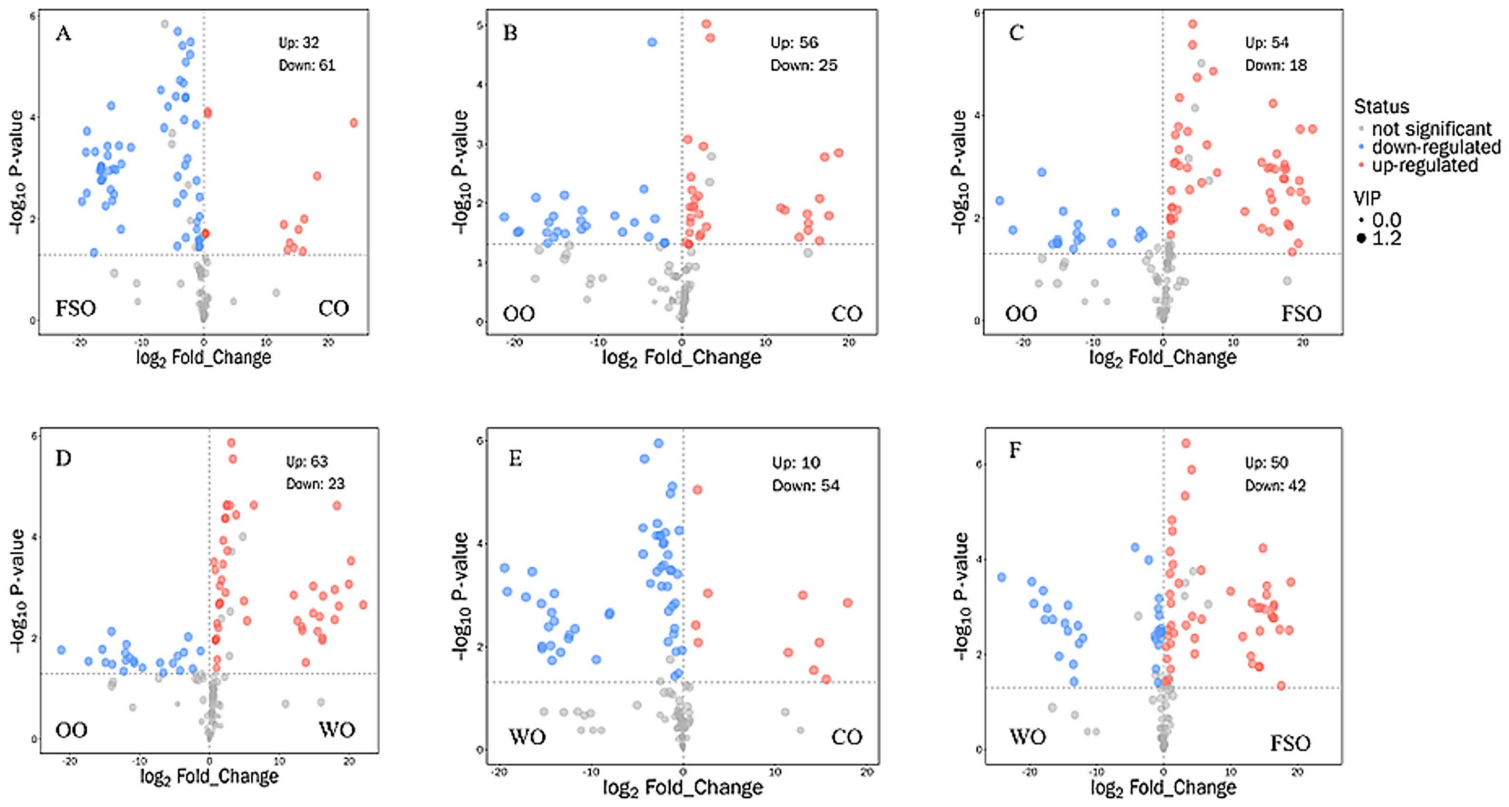
Volcano plots of differentially expressed volatile compounds between pairwise comparisons of the four vegetable oils. **(A)** Flaxseed oil vs. Camellia oil (FSO vs. CO); **(B)** Olive oil vs. Camellia oil (OO vs. CO); **(C)** Olive oil vs. Flaxseed oil (OO vs. FSO); **(D)** Olive oil vs. Walnut oil (OO vs. WO); **(E)** Walnut oil vs. Camellia oil (WO vs. CO); **(F)** Walnut oil vs. Flaxseed oil (WO vs. FSO).

A volcano plot is a graphical tool commonly used to display differences in gene expression, proteomics, and metabolomics data. It intuitively illustrates the significance and extent of differential expression by combining statistical significance (*p*-value) with fold change. In this study, a volcano plot was employed to visualize the differential expression of volatile components in four edible oils (WO, OO, FSO, CO). Figures 2A–F present the volcano plots of volatile components in these four vegetable oils. Figure 2A compares FSO with CO, identifying a total of 93 differentially expressed volatile components, with 32 upregulated and 61 downregulated. This figure indicates significant differences in the composition of volatile components between FSO and CO, with (E)-2-Penten-1-ol and Pentanoic acid, 2-methyl-, anhydride in flaxseed oil being the most significant differential volatile compounds. Notably, (E)-2-Penten-1-ol possesses a strong fruity or herbal aroma reminiscent of apples or bananas. Figure 2B compares OO with CO, screening a total of 81 differentially expressed volatile components, of which 56 were upregulated and 25 were downregulated. The results demonstrate significant differences in volatile components between OO and CO, with the number of upregulated components in OO being notably higher than the number of downregulated components. Figure 2C compares FSO with OO, identifying a total of 72 differentially expressed volatile components, among which 54 were upregulated and 18 were downregulated. This figure further confirms the differences in volatile components between FSO and OO, with a greater number of upregulated components in FSO, thereby substantiating the differences in their chemical compositions. Figure 2C compares FSO with OO, identifying a total of 72 differentially expressed volatile components, of which 54 were upregulated and 18 were downregulated. This figure further confirms the differences in volatile components between FSO and OO, highlighting a greater number of upregulated components in FSO, thereby substantiating the distinctions in their chemical compositions. Figure 2D compares OO with WO, revealing a total of 86 differentially expressed volatile components, with 63 upregulated and 23 downregulated. The results indicate significant differences in volatile components between OO and WO, with the number of upregulated components in OO being markedly higher than those that were downregulated. This suggests that OO may possess a greater abundance of volatile components that positively influence flavor, potentially enhancing its aroma and taste. Figure 2E contrasts WO with CO oil, identifying a total of 64 differentially expressed volatile components, including 10 that were upregulated and 54 downregulated. This figure delineates the differences in volatile components between WO and CO, indicating a higher number of downregulated components in CO. This may imply that CO has a simpler composition of volatile components or that these downregulated components contribute less to CO’s flavor profile. Figure 2F WO vs. FSO compares WO with FSO, revealing a total of 92 differentially expressed volatile components, of which 50 were upregulated and 42 were downregulated. These findings indicate significant differences in volatile components between WO and FSO, with a slightly greater number of components upregulated in WO than downregulated, potentially reflecting the richness of WO in specific volatile components. Through volcano plot analysis, this study successfully identified significantly differentially expressed volatile components in various edible oils. The variations in these components may have important implications for the flavor, stability, and nutritional value of edible oils. Minor deviations in individual volatile compound contents could be detected across replicate batches acquired from agricultural markets and manufacturing suppliers. Nevertheless, PCA and heatmap clustering results illustrated that such within-group fluctuations were negligible relative to the substantial metabolic gaps separating the four oil varieties. The consistent cold-chain logistics, limited short-term sealed storage and fully standardized pretreatment workflows adopted in this work successfully offset any analytical bias triggered by divergent retail storage conditions, proving that the unique volatile fingerprints identified in the present study stem from inherent chemical traits of each oil matrix rather than irregular sample storage backgrounds. Future research could further explore the specific mechanisms of action of these compounds and investigate methods to enhance the overall quality of edible oils by regulating their composition. Additionally, these differentially expressed volatile components may serve as potential candidate markers for assessing the quality of edible oils, providing a scientific basis for quality control and authenticity verification.

### 3.2 Identification and comparison of non-volatile metabolites

#### 3.2.1 Identification of non-volatile metabolites

Supplementary Figure S2 shows the TIC chromatograms of four oil samples acquired by LC–MS under negative (A1–D1) and positive (A2–D2) ionization modes. All samples exhibited clear baseline and well-separated chromatographic peaks, indicating that the established LC–MS method achieved satisfactory separation for non-volatile metabolites. Obvious differences in peak distribution and signal intensity were observed between the two ionization modes and among different oil samples. The complementary detection results from positive and negative modes ensured comprehensive profiling of metabolites in the four vegetable oils, laying a foundation for subsequent metabolite identification and classification.

**Figure 3:**
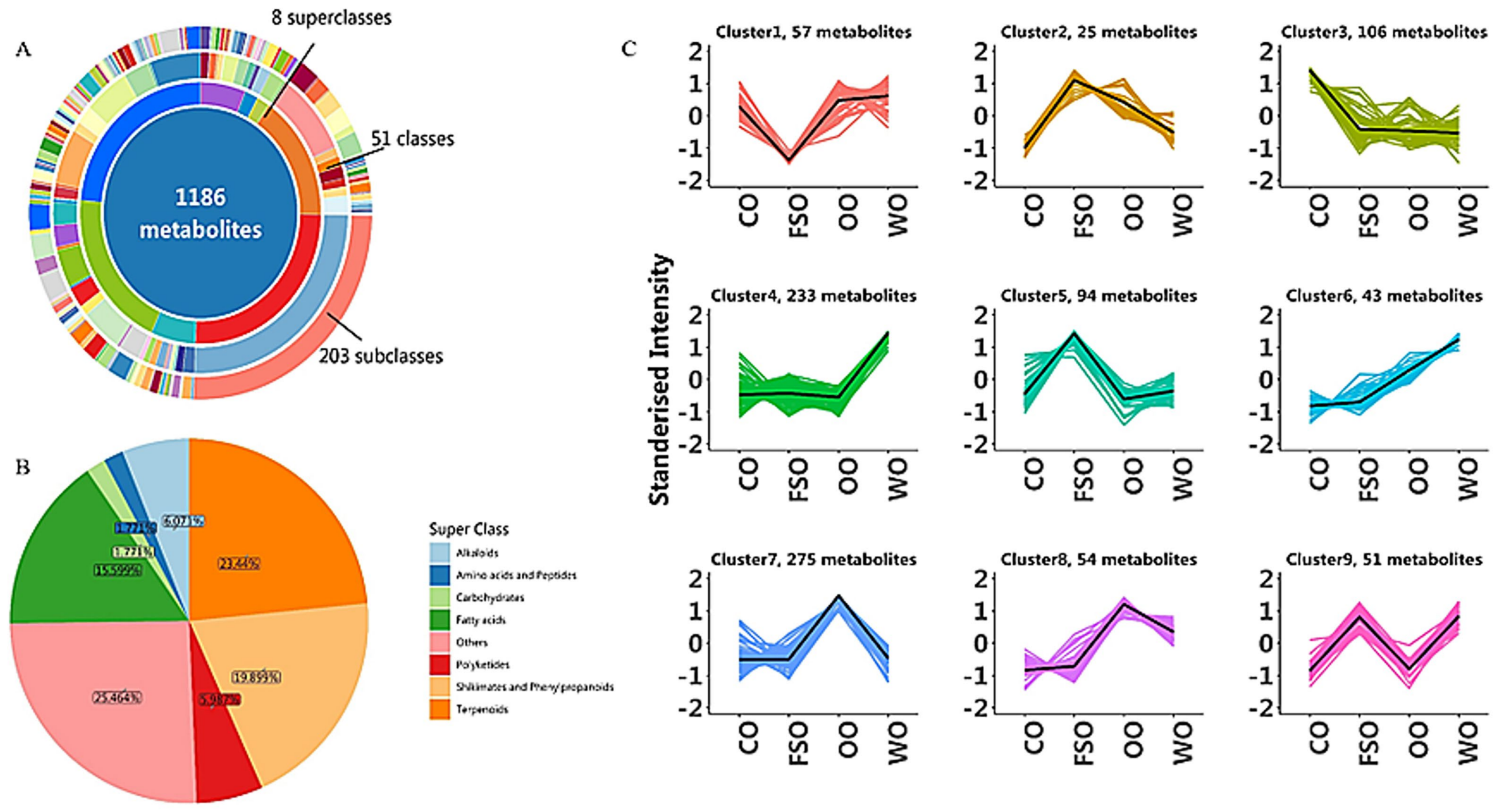
Comprehensive overview of non-volatile metabolite profiles in four vegetables oils: camellia oil (CO), flaxseed oil (FSO), olive oil (OO), and walnut oil (WO). **(A)** Circos plot; **(B)** Pie chart; **(C)** K-means clustering analysis of metabolite intensities across the four oil types.

Among the four vegetable oils analyzed, a total of 1,186 well-classified metabolites were detected as shown in the Figure 3A, which can be categorized into eight major groups: Alkaloids, Amino Acids and Peptides, Carbohydrates, Fatty Acids, Others, Polyketides, Shikimates and Phenylpropanoids, and Terpenoids in Figure 3B. Notably, Terpenoids constituted the largest proportion at 23.44%, suggesting that this sample may contain the greatest variety and quantity of terpenoid compounds. Other categories, including Fatty Acids, Others, and Carbohydrates, also represented substantial proportions at 15.59, 25.46, and 19.89%, respectively, highlighting the diversity of the metabolite composition in the oil sample. Each major category corresponds to distinct biochemical functions and metabolic pathways. For example, Fatty Acids are crucial for energy storage and metabolism, Amino Acids and Peptides are integral to protein synthesis, and Terpenoids frequently exhibit antioxidant properties, among other functions.

The primary metabolites of OO can be categorized into three main groups: first, fatty acids, which include oleic acid, linoleic acid, and palmitic acid, with oleic acid and linoleic acid present in higher concentrations; second, phenolic compounds, such as hydroxytyrosol and oleuropein aglycone derivatives (e.g., oleuropein aglycone); and third, triterpenes, including oleanolic acid and ursolic acid. The main metabolites of WO consist of fatty acids, such as linoleic acid and alpha-linolenic acid, both of which are present in significant amounts; phenolic compounds, including juglone and its derivatives, along with the detection of hydroxytyrosol; and phytosterols, such as beta-sitosterol, stigmasterol, and campesterol. The primary metabolites of FSO include fatty acids, particularly alpha-linolenic acid and linoleic acid, with alpha-linolenic acid being the most abundant, followed by linoleic acid; phenolic compounds, such as secoisolariciresinol and ferulic acid; in addition, lignan compounds have also been detected. The primary metabolites of CO comprise fatty acids, including oleic acid and linoleic acid; phenolic compounds, such as camelliasaponin and catechin; and triterpenes, including oleanolic acid and ursolic acid. In summary, olive oil and camellia oil predominantly contain oleic acid (a monounsaturated fatty acid) as their main fatty acid, whereas walnut oil and flaxseed oil are primarily composed of polyunsaturated fatty acids (linoleic acid and alpha-linolenic acid). Specific metabolites, such as hydroxytyrosol in olive oil, juglone in walnut oil, lignans in flaxseed oil, and saponins in camellia oil, are characteristic of each oil. The specific metabolites of each oil are closely related to their nutritional value and functional properties, including the antioxidant properties of olive oil and the anti-inflammatory effects of flaxseed oil, all of which are associated with these metabolites.

The nine clusters (Cluster 1–Cluster 9) exhibited distinct variation trends in standardized intensity, as shown in Figure 3C. For instance, Cluster4 (233 metabolites) showed an increase in intensity in WO, while Cluster7 (275 metabolites) peaked in OO, indicating that different oil samples (CO, FSO, OO, WO) have specific effects on metabolite accumulation. The clustering patterns can assist in screening characteristic metabolites of oil samples, linking oil varieties with metabolic regulation pathways. By combining the proportion of metabolite categories with clustering trends, Fatty acids are identified as key components (e.g., OO and CO are rich in monounsaturated fatty acids like oleic acid, while WO and FSO are prominent in polyunsaturated fatty acids). The proportion and clustering fluctuations of secondary metabolites such as phenolics (e.g., superclasses like Polyphenolics) may be related to the antioxidant and anti-inflammatory functions of the oils, providing a metabolomic basis for analyzing the differences in nutritional functions of oil samples.

#### 3.2.2 Differential analysis of non-volatile metabolites

**Figure 4:**
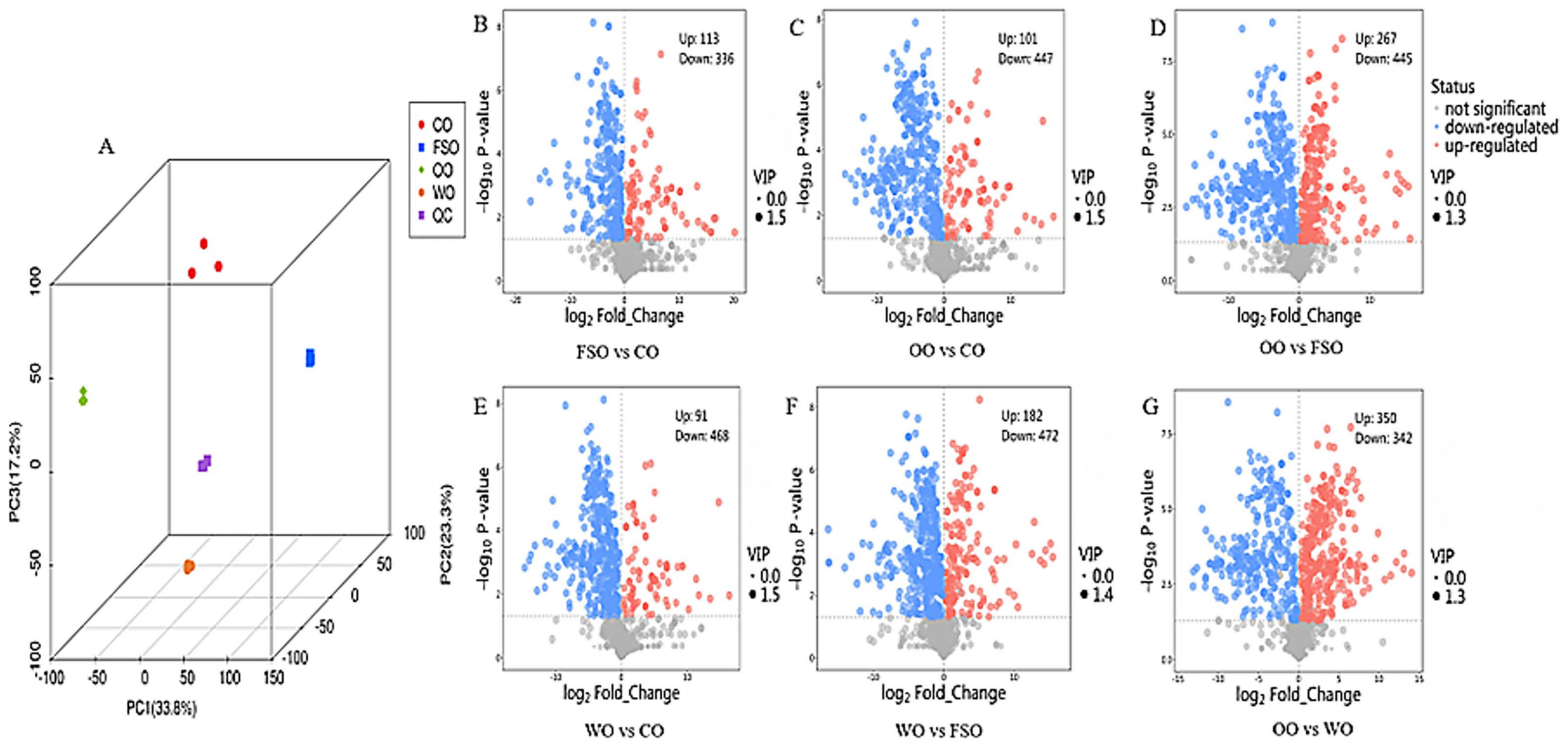
**(A)** Three - dimensional principal component analysis (3D–PCA) score plot of non-volatile metabolites in different vegetable oils and quality control (QC) samples; **(B–G)** Volcano plots showing the differential accumulation of non-volatile metabolites between different vegetable oil pairs.

PCA is a multivariate statistical method that constructs simplified mathematical models from high-dimensional complex data. It reveals the intrinsic structure among variables by extracting a limited number of principal components while retaining the maximum amount of information from the original dataset. In this study, PCA was performed on the metabolite content of four vegetable oils (WO, OO, FSO, CO) and quality control samples (QC), with the results illustrated in Figure 4A. The first principal component (PC1), the second principal component (PC2), and the third principal component (PC3) accounted for 33.8, 23.3, and 17.2% of the variance in the dataset, respectively. PC1 explains 33.8% of the variance, indicating that it is the primary axis for distinguishing between different oil types. In the score plot, QC samples are located in the central region, demonstrating that the analytical system is stable and exhibits good reproducibility. The four vegetable oil samples display a clear separation trend along the directions of PC1, PC2, and PC3, indicating significant differences in metabolite composition among the various oil types. Furthermore, samples within the same oil type cluster closely together, reflecting minimal biological variation within the group and confirming the reliability of the experimental data. Minor differences in several non-volatile metabolites were detected across batches from different suppliers, but multivariate analysis confirmed that gaps between oil matrices were far larger than within-group deviations. Uniform cold-chain delivery, dark cold storage and standardized oil extraction eliminated disturbances from inconsistent commercial storage, proving the observed metabolic differences originate from inherent traits of each oil rather than storage-related interference.

To further screen characteristic metabolites responsible for group differences, pairwise partial least squares-discriminant analysis (PLS-DA) was applied. All six pairwise PLS-DA models were validated via 200 permutation tests (Supplementary Figure S1). The intercepts of the Q^2^(cum) regression lines for all comparisons were negative, ranging from −0.38 to −0.29. Meanwhile, all permuted Q^2^ values were lower than the original Q^2^ value at the correlation coefficient of 1.0. These results verified that none of the PLS-DA models suffered from overfitting, and the metabolic discrepancies among the four oils were statistically reliable rather than caused by random variation. The identified differential metabolites mainly included phenolic compounds, tocopherols, phytosterols and diverse lipid derivatives, which could act as potential biomarkers to differentiate the four vegetable oils.

This study employed a VIP threshold greater than 1 and a *p*-value less than 0.05 as the screening criteria for identifying differential metabolites. Table 1 presents comparative data on metabolite differences among various vegetable oil groups (e.g., WO vs. FSO, OO vs. WO). According to the Cpd diff data (down-regulated differential metabolites), significant differences exist in the number of differential metabolites across the different vegetable oil groups, with the OO vs. FSO group exhibiting the highest number (712 types) and the FSO vs. CO group the lowest (449 types). This indicates a high degree of differentiation in the metabolomic characteristics between olive oil and flaxseed oil, whereas the metabolomic profiles of flaxseed oil and camellia oil are relatively similar. This variation is attributable to the inherent characteristics of the vegetable oils. Different vegetable oils display varying degrees of metabolite differences, which stem from differences in fatty acid composition and secondary metabolite synthesis pathways. For instance, olive oil is abundant in monounsaturated fatty acids, such as oleic acid, and phenolic compounds, such as hydroxytyrosol, while flaxseed oil primarily contains polyunsaturated fatty acids, such as *α*-linolenic acid, along with lignans. The synthesis pathways of metabolites in these two oils differ significantly, leading to a substantial number of differential metabolites. By integrating Cpd diff up and Cpd diff down analyses, we can effectively examine the regulatory trends of metabolites across different groups. Taking the WO vs. FSO group as an illustrative example, we observe that the number of down-regulated differential metabolites (472) significantly exceeds that of up-regulated metabolites (182). This suggests that, in comparison to flaxseed oil, walnut oil contains a greater number of metabolites with decreased concentrations. This phenomenon may be attributed to the distinct metabolic synthesis preferences associated with walnut oil and flaxseed oil. Notably, walnut oil exhibits a unique pattern of synthesis and accumulation for phenolic compounds, such as juglone, and specific phytosterols. These compounds may either compete with or synergistically influence the synthesis of metabolites like α-linolenic acid found in flaxseed oil, resulting in an imbalance in the regulatory direction of differential metabolites. The volcano plots (Figures 4BG) utilize log₂Fold_Change (x-axis, representing the fold change in metabolite expression between the two groups) and -log₁₀*p*-value (y-axis, indicating the significance of differences) to illustrate the metabolite differences among various vegetable oil groups (e.g., FSO vs. CO, OO vs. WO, etc.). In the volcano plot for the OO vs. WO group (Figure 4G), the red scatter points (representing up-regulated metabolites) are predominantly clustered in the high fold change region. This concentration indicates that the metabolite differences between olive oil and walnut oil are both numerous and statistically significant, thereby reinforcing the distinct metabolic characteristics observed between these groups.

**Table 1:** Significantly different metabolites in each comparison group (TOP 15).

MS2name	m/z	rt	Super class	VIP	*p*-value	FC
WO vs FSO						
Solasodine base + 2H, O-Hex-Hex-Hex-Pen	1034.5706	249.8	Alkaloids	1.3613	0.0006	46352.8368
24,27-dibenzyl-12,21-diisobutyl-9-isopropyl-15,18-disec-butyl-1,7,10,13,16,19,22,25,28-nonazatricyclo[28.3.0.03,7]tritriacontane-2,8,11,14,17,20,23,26,29-nonone	1038.6353	274.8	Amino acids and Peptides	1.3155	0.0002	37309.0508
Zingiberensis saponin I	1069.526	266.1	Terpenoids	1.3613	0.0004	22819.2008
Tomatoside_A	1083.5484	266.1	Terpenoids	1.3612	0.0010	19692.5857
Ampeloside_Bf1	1115.5381	239.9	Terpenoids	1.3613	0.0008	13726.2747
Melongoside_P	1067.5654	254.5	Terpenoids	1.3613	0.0000	7478.4064
Cyclolinopeptide_I	1068.5005	252.3	Amino acids and Peptides	1.2977	0.0005	6039.7404
Cyclolinopeptide_H	1082.5161	257.1	Amino acids and Peptides	1.3565	0.0002	1236.3572
Pipereicosalidine	374.3408	332.3	Alkaloids	1.3607	0.0102	1029.5223
9-methoxy-4-(2-methylbut-3-en-2-yl)furo[3,2-g]chromen-7-one [IIN-based on]	569.2133	236	Shikimates and Phenylpropanoids	1.3607	0.0077	614.9947
(3R,4R)-4-(1,3-benzodioxol-5-ylmethyl)-3-[(3,4,5-trimethoxyphenyl)methyl]tetrahydrofuran-2-one	401.158	223.1	Shikimates and Phenylpropanoids	1.3612	0.0007	381.1271
5-nonadecylbenzene-1,3-diol	375.326	344.6	Polyketides	1.3376	0.0170	248.6131
Gomisin A	417.1894	211.8	Shikimates and Phenylpropanoids	1.3607	0.0007	196.8576
formyl_2Z,4E,6Z-decatrienoate	181.1217	309.5	Fatty acids	1.2068	0.0223	183.9670
Antheraxanthin	585.4273	308.5	Terpenoids	1.3524	0.0032	154.7220
Androstendione	287.1996	226.5	Terpenoids	1.3062	0.0000	149.0175
OO vs WO						
2-Hydroxydodeca-5,8-dienoylcarnitine	356.2421	272.5	Organic acids and derivatives	1.3085	0.0013	16,947.0364
Phosphatidylserine (1-palmitoyl, 2-palmitoleoyl)	734.4869	235.8	Lipids and lipid - like molecules	1.3085	0.0003	8,955.2063
PI(18:1/18:1)	861.547	308.2	Fatty acids	1.3085	0.0010	8,643.1870
(S)-Autumnaline	374.1951	237.4	Alkaloids	1.2441	0.0006	4,564.2739
Debromoaplysiatoxin	593.3336	236.3	Polyketides	1.3085	0.0022	1,700.1017
Liquidambaric acid	455.3442	294.6	Terpenoids	1.2478	0.0042	426.3084
4,5,5,15,15-pentamethyl-3,8,14-trioxatetracyclo[11.4.0.02,6.07,12]heptadeca-1(13),2(6),7(12),10,16-pentaen-9-one	311.1285	228.3	Shikimates and Phenylpropanoids	1.3083	0.0003	183.6229
Platyphylline	360.1795	225	Alkaloids	1.3085	0.0007	172.5337
2-Methoxy-4-{(2S,3R)-7-methoxy-3-methyl-5-[(1E)-1-propen-1-yl]-2,3-dihydro-1-benzofuran-2-yl}phenol	344.1845	231.4	Shikimates and Phenylpropanoids	1.2436	0.0013	164.1790
Narcotine	452.1146	238.3	Alkaloids	1.2568	0.0023	155.8442
sinomenine	330.169	227.6	Alkaloids	1.2889	0.0009	149.9061
10-hydroxy-4-isopropyl-1-(2-methylpropylidene)-4H-pyrazino[2,1-b]quinazoline-3,6-dione	326.1477	231	Alkaloids	1.3073	0.0000	130.0553
O-Oxalylhomoserine	192.0505	191.2	Amino acids and Peptides	1.3084	0.0007	130.0131
Methyl_(7Z,9Z,9’Z)-6′-apo-y-caroten-6′-oate	473.3435	289.2	Terpenoids	1.1848	0.0127	113.4759
Pavetannin_A2	577.1301	253	Shikimates and Phenylpropanoids	1.1957	0.0000	112.9648
FSO vs CO						
(3S,6R,12S)-6-benzyl-3-isobutyl-9-[6-(oxiran-2-yl)-6-oxo-hexyl]-1,4,7,10-tetrazabicyclo[10.4.0]hexadecane-2,5,8,11-tetrone	569.3295	287	Amino acids and Peptides	1.4722	0.0108	105,314.2637
Anthranoyllycoctonine	587.3411	286.3	Alkaloids	1.4723	0.0109	87,417.4654
2,4-Dihydroxybutanoic acid	101.0243	16.7	Fatty acids	1.4702	0.0298	59,641.9699
2-Ketobutyric acid	101.0243	16.7	Fatty acids	1.4702	0.0298	59,641.9699
Flurandrenolide	437.2282	262	Terpenoids	1.4704	0.0226	31,781.8937
Denticulaflavonol	559.3099	277	Shikimates and Phenylpropanoids	1.4721	0.0188	23,997.7183
LPA(20:5)	457.2417	277.3	Fatty acids	1.4722	0.0138	11,691.3147
COSTUNOLIDE	231.1385	264.6	Terpenoids	1.4722	0.0011	10,159.7280
Goniothalenol	231.0658	246.1	Shikimates and Phenylpropanoids	1.4319	0.0157	2,667.5404
Liquidambaric acid	455.3442	294.6	Terpenoids	1.4724	0.0088	1,483.2535
(5S,9R)-14-(hydroxymethyl)-5,9-dimethyl-tetracyclo[11.2.1.01,10.04,9]hexadec-14-ene-5-carboxylic acid	317.2115	273.5	Terpenoids	1.4700	0.0016	1,150.9391
4-[2-[(1R,4aS,5R,8aS)-5-(hydroxymethyl)-5,8a-dimethyl-2-methylene-decalin-1-yl]ethyl]-2H-furan-5-one	317.2115	273.5	Terpenoids	1.4700	0.0016	1,150.9391
(8E)-10-hydroxy-5,9,12,13,16-pentamethyl-4-oxatricyclo[10.3.1.03,5]hexadeca-1(15),8-dien-2-one	317.2115	273.5	Terpenoids	1.4700	0.0016	1,150.9391
*N*-Linoleoyl Lysine	409.3415	256.2	Organic acids and derivatives	1.4729	0.0064	625.9510
Paulownin	369.0974	232.9	Shikimates and Phenylpropanoids	1.4697	0.0044	540.1301
OO vs CO						
Anthranoyllycoctonine	587.3411	286.3	Alkaloids	1.4878	0.0109	87,167.4916
Denticulaflavonol	559.3099	277	Shikimates and Phenylpropanoids	1.4874	0.0188	23,929.0959
LPA(20:5)	457.2417	277.3	Fatty acids	1.4875	0.0138	11,657.8829
17-[2,3-dihydroxy-6-methyl-6-[3,4,5-trihydroxy-6-(hydroxymethyl)oxan-2-yl]oxyheptan-2-yl]-2,3,14-trihydroxy-10,13-dimethyl-2,3,4,5,9,11,12,15,16,17-decahydro-1H-cyclopenta[a]phenanthren-6-one	660.3935	299.5	Terpenoids	1.4882	0.0000	28,644.4688
(3S,6R,12S)-6-benzyl-3-isobutyl-9-[6-(oxiran-2-yl)-6-oxo-hexyl]-1,4,7,10-tetrazabicyclo[10.4.0]hexadecane-2,5,8,11-tetrone	569.3295	287	Amino acids and Peptides	1.4337	0.0108	4,684.6953
N-arachidonoyl-glutamate	434.2887	265.4	Organic acids and derivatives	1.4843	0.0304	2,079.5068
Bufalin	385.2377	292.1	Terpenoids	1.4726	0.0013	894.3726
[(2E,6E)-8-(2,6-dihydroxy-4-methyl-phenyl)-2,6-dimethyl-1-(3-methylbut-2-enyl)octa-2,6-dienyl] acetate	385.2377	292.1	Terpenoids	1.4726	0.0013	894.3726
12-HEPE	317.2116	257.4	Fatty acids	1.4877	0.0014	718.7566
Prexanthoperol	315.1944	229.2	Terpenoids	1.4669	0.0028	493.4610
Paulownin	369.0974	232.9	Shikimates and Phenylpropanoids	1.4870	0.0044	231.3426
9-hydroxy-7-isopropyl-1,4a-dimethyl-2,3,4,9,10,10a-hexahydrophenanthrene-1-carboxylic acid	315.1958	264.9	Terpenoids	1.4877	0.0012	204.5542
all-trans-5,6-Epoxyretinoic acid	315.1958	264.9	Terpenoids	1.4877	0.0012	204.5542
Liquidambaric acid	455.3442	294.6	Terpenoids	1.3947	0.0086	125.2723
Goniothalenol	231.0658	246.1	Shikimates and Phenylpropanoids	1.4862	0.0159	122.4004
OO vs FSO						
PI(18:1/18:1)	861.547	308.2	Fatty acids	1.2773	0.0370	54,665.8811
Solasodine base + 2H, O-Hex-Hex-Hex-Pen	1034.5706	249.8	Alkaloids	1.2781	0.0006	46,277.0241
Cyclolinopeptide_I	1068.5005	252.3	Amino acids and Peptides	1.2567	0.0005	31,752.7192
Zingiberensis saponin I	1069.526	266.1	Terpenoids	1.2781	0.0004	22,781.8787
Tomatoside_A	1083.5484	266.1	Terpenoids	1.2781	0.0010	19,660.3773
24,27-dibenzyl-12,21-diisobutyl-9-isopropyl-15,18-disec-butyl-1,7,10,13,16,19,22,25,28-nonazatricyclo[28.3.0.03,7]tritriacontane-2,8,11,14,17,20,23,26,29-nonone	1038.6353	274.8	Amino acids and Peptides	1.2697	0.0002	16,305.7407
Ampeloside_Bf1	1115.5381	239.9	Terpenoids	1.2781	0.0008	13,703.8246
1–18:3–2-18:3-phosphatidylinositol	853.4846	290.7	Lipids and lipid-like molecules	1.2777	0.0121	13,179.4450
Melongoside_P	1067.5654	254.5	Terpenoids	1.2781	0.0000	7,466.1751
Cyclolinopeptide_H	1082.5161	257.1	Amino acids and Peptides	1.2689	0.0002	5,312.4904
5-nonadecylbenzene-1,3-diol	375.326	344.6	Polyketides	1.2571	0.0169	2,051.3009
FR900359	1002.5354	259	Amino acids and Peptides	1.2770	0.0241	1,502.4102
Pipereicosalidine	374.3408	332.3	Alkaloids	1.2620	0.0102	474.5502
(3R,4R)-4-(1,3-benzodioxol-5-ylmethyl)-3-[(3,4,5-trimethoxyphenyl)methyl]tetrahydrofuran-2-one	401.158	223.1	Shikimates and Phenylpropanoids	1.2773	0.0007	454.7093
1,4-Androstadiene-3,17-dione	285.1839	225.1	Terpenoids	1.2654	0.0008	264.0892
WO vs CO						
Anthranoyllycoctonine	587.3411	286.3	Alkaloids	1.4630	0.0109	87,310.2927
17-[2,3-dihydroxy-6-methyl-6-[3,4,5-trihydroxy-6-(hydroxymethyl)oxan-2-yl]oxyheptan-2-yl]-2,3,14-trihydroxy-10,13-dimethyl-2,3,4,5,9,11,12,15,16,17-decahydro-1H-cyclopenta[a]phenanthren-6-one	660.3935	299.5	Terpenoids	1.4633	0.0000	28,691.3953
LPA(20:5)	457.2417	277.3	Fatty acids	1.4627	0.0138	11,676.9813
(3S,6R,12S)-6-benzyl-3-isobutyl-9-[6-(oxiran-2-yl)-6-oxo-hexyl]-1,4,7,10-tetrazabicyclo[10.4.0]hexadecane-2,5,8,11-tetrone	569.3295	287	Amino acids and Peptides	1.4038	0.0108	3,528.7750
5,6,16-Trihydroxygrayanotox-10-en-3-yl_hexopyranoside	499.2888	290.3	Shikimates and Phenylpropanoids	1.4170	0.0033	2,655.1802
Bufalin	385.2377	292.1	Terpenoids	1.4546	0.0013	622.5478
[(2E,6E)-8-(2,6-dihydroxy-4-methyl-phenyl)-2,6-dimethyl-1-(3-methylbut-2-enyl)octa-2,6-dienyl] acetate	385.2377	292.1	Terpenoids	1.4546	0.0013	622.5478
12-HEPE	317.2116	257.4	Fatty acids	1.4596	0.0014	383.0157
Denticulaflavonol	559.3099	277	Shikimates and Phenylpropanoids	1.3513	0.0189	319.2466
Paulownin	369.0974	232.9	Shikimates and Phenylpropanoids	1.4095	0.0042	142.4077
Flurandrenolide	437.2282	262	Terpenoids	1.4205	0.0229	133.2857
(5S,9R)-14-(hydroxymethyl)-5,9-dimethyl-tetracyclo[11.2.1.01,10.04,9]hexadec-14-ene-5-carboxylic acid	317.2115	273.5	Terpenoids	1.4438	0.0015	119.6488
4-[2-[(1R,4aS,5R,8aS)-5-(hydroxymethyl)-5,8a-dimethyl-2-methylene-decalin-1-yl]ethyl]-2H-furan-5-one	317.2115	273.5	Terpenoids	1.4438	0.0015	119.6488
(8E)-10-hydroxy-5,9,12,13,16-pentamethyl-4-oxatricyclo[10.3.1.03,5]hexadeca-1(15),8-dien-2-one	317.2115	273.5	Terpenoids	1.4438	0.0015	119.6488
9-hydroxy-7-isopropyl-1,4a-dimethyl-2,3,4,9,10,10a-hexahydrophenanthrene-1-carboxylic acid	315.1958	264.9	Terpenoids	1.4555	0.0012	117.4153

Table 1 presents the top 15 significantly different metabolites across six control groups (WO vs. FSO, OO vs. WO, FSO vs. CO, OO vs. CO, OO vs. FSO, WO vs. CO). Notably, the VIP values are mostly above 1.2 (indicating significant contributions to the differences), *p*-values are predominantly ≤0.05 (demonstrating statistical significance), and the FC values vary greatly (ranging from hundreds to over one hundred thousand). The primary supercategories include Terpenoids, Alkaloids, Amino acids and Peptides, and Fatty acids, with distinct differences in the dominant metabolite categories and the degree of variation across the different control groups. Among them, the FSO vs. CO control group exhibits the highest FC value of 105314.2637, corresponding to the metabolite “(3S,6R,12S)-6-benzyl-3-isobutyl-9-[6-(oxiran-2-yl)-6-oxo-hexyl]-1,4,7,10-tetrazabicyclo [10.4.0] hexadecane-2,5,8,11-tetrone,” which falls under the category of Amino acids and Peptides. In the comparisons between WO vs. FSO (5 types), FSO vs. CO (4 types), OO vs. CO (4 types), and WO vs. CO (5 types), terpenoids are the predominant category, with a total occurrence far exceeding that of other categories. Terpenoids, a diverse class of natural compounds, play a significant role in the nutritional and functional properties of vegetable oils such as flaxseed oil, camellia oil, walnut oil, and olive oil. These compounds are synthesized from isoprene units and contribute to the oxidative stability, flavor, and health benefits of these oils. Olive oil is one of the most extensively studied vegetables oils regarding terpenoid compounds, with its terpenoid components primarily derived from the fruit of the olive tree (*Olea europaea* L.). These components include monoterpenes, sesquiterpenes, and triterpenes, among which squalene is the most characteristic terpenoid in olive oil. Squalene serves as a precursor for cholesterol synthesis and possesses strong antioxidant properties, capable of scavenging free radicals and protecting cell membrane lipids from oxidative damage (17). Monoterpenes, which are mainly volatile components such as limonene, *α*-pinene, and citronellol, impart a unique fruity aroma to olive oil while also exhibiting anti-inflammatory and antibacterial activities (18). The terpene compounds in camellia oil are primarily concentrated in the non-saponifiable fraction, characterized by triterpenes and their glycosides (saponins), which are the core active components. Camelliaside, a characteristic component, mainly consists of oleanane-type triterpenoid aglycones (such as ursolic acid and oleanolic acid), with sugar chains composed of glucose, galactose, and others. Camelliaside exhibits significant anti-inflammatory and lipid-lowering activities (19). The volatile components contain a small amount of *β*-Caryophyllene, which imparts a subtle floral aroma to camellia oil while also exhibiting anti-inflammatory activity (by activating CB₂ receptors) (20). Walnut oil contains α-pinene, β-pinene, and limonene, with their concentrations varying according to the variety and maturity of the walnuts. These compounds are the main contributors to the ‘nutty aroma’ of walnut oil (21). Additionally, walnut oil contains triterpenoid compounds, such as β-sitosterol, which, despite being a sterol, is an important antioxidant component (22). The content of terpenoids in flaxseed oil is relatively low, primarily consisting of sesquiterpenes and a small amount of triterpenes. These terpenoids are not only important markers of the flavor and quality of vegetable oils, but their biological activities also provide a scientific basis for the ‘health functions’ of vegetable oils. Future research could focus on the synergistic effects of terpenoids and fatty acids, as well as the impact of processing techniques on the retention rates of terpenoids, providing references for the development of functional vegetable oils.

In WO vs. FSO, terpenoids are predominant, followed by alkaloids, and amino acids and peptides. Flaxseed oil contains a high content of saponins (e.g., Zingiberensis saponin I and Tomatoside_A) and cyclopeptides (e.g., Cyclolinopeptide_H and Cyclolinopeptide_I). Saponin compounds have various biological functions in plants, including antioxidant, anti-inflammatory, and immunomodulatory effects. Flaxseed cyclopeptides (CLPs) are a group of cyclic peptides derived from flaxseed oil, recognized for their unique structural and functional properties. The structural diversity of CLPs is notable, with up to 15 cyclic peptides identified in freshly prepared flaxseed oil using advanced analytical techniques such as RP-HPLC coupled with Orbitrap mass spectrometry. These peptides, including CLP-A, CLP-B, CLP-C, CLP-D, CLP-E, CLP-F, CLP-G, CLP-K, CLP-L, CLP-M, CLP-N, CLP-O, CLP-P, and CLP-T, exhibit varying stability and oxidation patterns, which are critical for assessing the quality of flaxseed oil (23). For example, CLP-E is often used as an indicator of bitterness in flaxseed oil, while the presence of methionine-containing peptides and their oxidation products, such as methionine sulfoxide and methionine sulfone, can provide insights into the oxidative stability of the oil. The stability of CLPs has been evaluated under different conditions, revealing that certain peptides (24), such as CLP-O, CLP-B, and CLP-N, degrade rapidly at room temperature, while others, like CLP-A and CLP-C, remain stable over extended periods. Both CLH and CLI exhibit significant antioxidant activity, effectively inhibiting the free radicals generated during the oxidation process of oils, thereby extending the shelf life of flaxseed oil (25). This information is crucial for optimizing storage and processing conditions to preserve the quality of flaxseed oil.

The categories covered in OO vs. WO include Organic acids and derivatives, Lipids, and Alkaloids, among others. The maximum FC value is 16947.0 for 2-Hydroxydodeca-5,8-dienoylcarnitine. Walnut oil contains a high concentration of Alkaloids (such as Altretamine and Flazine) and Shikimates and Phenylpropanoids (such as Cinnamic acids and derivatives like Sinapoylmalate and Disinapoyl Hexoside), which aligns with the findings of most studies confirming the richness of walnut oil in tocopherols, phytosterols, and carotenoids (26). The significant difference in PI(18:1/18:1) (phosphatidylinositol, containing diacyl groups) between walnut oil and olive oil (with an FC value as high as 8643.1870) is a distinguishing substance. It is also the substance with the highest FC value in the OO vs. FSO groups.

In the comparison of SO vs. CO, amino acids and peptides, alkaloids, and fatty acids emerge as the primary categories. Notably, 2,4-dihydroxybutanoic acid and 2-ketobutyric acid are the most significantly different fatty acids, classified as short-chain hydroxy acids, which typically function as intermediates or secondary metabolites in specific metabolic pathways. In the comparison of OO vs. CO, alkaloids, shikimates, phenylpropanoids, and terpenoids are predominant. The maximum value of the fold change (FC) is 87167.5, attributed to anthranoyllycoctonine (alkaloids). Denticulaflavonol, a flavonoid, demonstrates significant differences in both FSO vs. CO and OO vs. CO, exhibiting moderate antitumor activity against various cancer cell lines, including lung cancer (A549), colon cancer (HT-29), cervical cancer (HeLa), and human leukemia (HL-60) cell lines (27). In the WO vs. CO comparison, alkaloids, terpenoids, and fatty acids are again predominant. The maximum FC value recorded is 87310.3 (anthranoyllycoctonine, alkaloids), while the minimum *p*-value is 0.0000 (17-substituted steroids, terpenoids). Steroids within the terpenoid category are significantly different substances in the WO vs. CO comparison, including 17-[2,3-dihydroxy-6-methyl-6-[3,4,5-trihydroxy-6-(hydroxymethyl)oxan-2-yl]oxyheptan-2-yl]-2,3,14-trihydroxy-10,13-dimethyl-2,3,4,5,9,11,12,15,16,17-decahydro-1H-cyclopenta[a]phenanthren-6-one and bufalin.

### 3.3 Integrated summary of characteristic volatile and non-volatile biomarkers

The utilization of Partial Least Squares-Discriminant Analysis (PLS-DA) models with Variable Importance in Projection (VIP) scores greater than 1 allows for the rigorous screening of differential metabolites that are exclusively up-regulated in specific vegetable oils, as shown as Table 2. This multi-dimensional approach effectively distinguishes WO, FSO, OO, and CO by leveraging their unique endogenous secondary metabolites and volatile flavor compounds, thereby establishing a robust framework for detecting adulteration (28).

**Table 2:** Integrated summary of characteristic volatile and non-volatile biomarkers in four vegetable oils.

Biomarker type	Oil type	Compound	Superclass	VIP
Non-volatile biomarkers	WO	Neoraunone	Isoflavanones	1.464
		Methylophiopogonone A	Chromones	1.463
		1–22:0–2-18:1-phosphatidylserine	-	1.463
		Phosphatidylserine (1-palmitoyl, 2-palmitoleoyl)	Hydroxy fatty acids	1.463
		Pavetannin_A2	Proanthocyanins	1.463
		1–24:0–2-18:2-phosphatidylserine	-	1.463
		Rehmannic acid	Chalcones	1.462
		(S)-Autumnaline	Isoquinoline alkaloids	1.429
	FSO	Solasodine base + 2H, O-Hex-Hex-Hex-Pen	Steroidal alkaloids	1.473
		Ampeloside_Bf1	Furostane steroids	1.473
		Melongoside_P	Furostane steroids	1.472
		Zingiberensis saponin I	Spirostane steroids	1.472
		FR900359	Cyclic peptides	1.472
	OO	[6-hydroxy-7-methyl-8-oxo-3-[(E)-prop-1-enyl]-5,6-dihydro-1H-isochromen-7-yl] 4-hydroxy-2-methoxy-6-methyl-benzoate	Azaphilones	1.488
		Loratadine	Alkaloids	1.488
		Methyl (2S,3R,4S)-3-ethenyl-4-[(Z)-3-[(2S,3R,4R)-3-ethenyl-5-methoxycarbonyl-2-.]-3,4-dihydro-2H-pyran-5-carboxylate]	Secoiridoid monoterpenoids	1.488
	CO	17-[2,3-dihydroxy-6-methyl-6-[3,4,5-trihydroxy-6-(hydroxymethyl)oxan-2-yl]oxyheptan-2-yl]-.-1H-cyclopenta[a]phenanthren-6-one	Ecdysteroids	1.488
		Anthranoyllycoctonine	Terpenoid alkaloids	1.488
		LPA(20:5)	Glycerophosphates	1.487
Volatile biomarkers	WO	1-Heptanol	Alcohols	1.231
	OO	1-Penten-3-one	Ketones	100
	FSO	1-Pentanol	Alcohols	2.097
		1-Penten-3-ol	Alcohols	1.466
		Benzyl alcohol	Alcohols	2.636
		2-Heptanol	Alcohols	1.222
		2-Pentenal, (E)-	Aldehydes	1.485
		Hexanoic acid	Acids	2.185
		β-Myrcene	Terpenoids	1.117
		2-Heptanone	Ketones	1.252
	CO	Hexanal	Aldehydes	17.03

VIP values are retained to three decimal places, representing the maximum variable importance in projection score from all pairwise OPLS-DA comparisons. Oil-specific biomarkers are defined as metabolites exclusively up-regulated in one oil and not identified as differential markers in the other three oils. “–” indicates unassigned chemical subclass.

Non-volatile biomarkers provide the most definitive evidence for botanical origin due to their genetic determination and stability. For walnut oil, the exclusive presence of multiple phosphatidylserine lipids, with VIP values around 1.46, alongside isoflavanones and proanthocyanins, reflects the distinct phospholipid composition of walnut kernels (28). Phosphatidylserine serves as a critical lipid class marker, differentiating WO from other seed oils that may lack this specific phospholipid profile (29). In contrast, flaxseed oil is characterized by a high concentration of steroidal alkaloids, furostane steroids, and spirostane steroids, with VIP values exceeding 1.47 (29). These steroidal compounds are typical endogenous secondary metabolites of *Linum usitatissimum*, offering strong species specificity that is difficult to replicate with common adulterants such as sunflower or rapeseed oil (30). Olive oil authentication relies heavily on secoiridoid monoterpenoids and azaphilones, with VIP values reaching up to 1.488 (31). Secoiridoids, such as oleuropein derivatives, are well-documented signature components of olive fruit and are widely recognized as primary quality indicators for virgin olive oil, distinguishing it from refined oils and other vegetable matrices (32). Camellia oil is uniquely identified by ecdysteroids and terpenoid alkaloids, which serve as core tracers for traceability given their characteristic presence in Camellia plants (33). The detection of these non-volatile markers via high-resolution mass spectrometry ensures high specificity, as these compounds are not typically found in the major adulterant oils like soybean or corn oil (34).

Volatile biomarkers, while fewer in number for some oils, provide complementary information regarding oxidative status and enzymatic activity. Flaxseed oil exhibits the largest number of unique volatile biomarkers, including benzyl alcohol (VIP = 2.636) and hexanoic acid (VIP = 2.185) (28). These compounds are primarily derived from the oxidative degradation of polyunsaturated fatty acids and amino acid metabolism, constituting the unique flavor profile of FSO (35). The high VIP score of benzyl alcohol indicates its significant contribution to the discriminant model, likely resulting from the breakdown of phenylalanine during seed processing. Conversely, olive oil is marked by 1-penten-3-one (VIP = 100.000), a characteristic degradation product of linoleic acid catalyzed by lipoxygenase (14). This extremely high VIP value underscores the critical role of lipoxygenase pathways in defining the sensory and chemical identity of virgin olive oil (36). Camellia oil is distinguished by hexanal (VIP = 17.030), a typical aldehyde product from the auto-oxidation of oleic and linoleic acids (37). Hexanal is a primary marker of lipid oxidation in high-oleic oils, and its elevated VIP score reflects the specific fatty acid composition of camellia seed (35). Walnut oil is identified by 1-heptanol (VIP = 1.231), which is closely associated with the oxidative metabolism of long-chain unsaturated fatty acids (28). The presence of these volatile markers, detected via techniques such as headspace gas chromatography–mass spectrometry (HS-GC–MS), provides a rapid assessment tool for authenticity that correlates with sensory propertie (38).

The integration of these volatile and non-volatile markers into a multi-dimensional identification system significantly enhances the accuracy of oil authentication. Traditional methods relying solely on fatty acid profiles are often insufficient due to the similarities between certain oils, such as camellia and olive oil, both of which are rich in oleic acid (39). By incorporating specific secondary metabolites like ecdysteroids for camellia oil and secoiridoids for olive oil, the risk of misclassification is substantially reduced. Furthermore, the use of PLS-DA models allows for the visualization of complex metabolic data, enabling clear separation of oil types based on their unique chemical fingerprints (40). This approach is particularly valuable for detecting sophisticated adulteration strategies where lower-quality oils are blended with high-value varieties (41). The high VIP values observed for key markers, such as 1-penten-3-one in olive oil and benzyl alcohol in flaxseed oil, demonstrate their robustness as diagnostic tools (28). Future developments in targeted metabolomics will likely focus on the quantitative validation of these markers, ensuring their reliability across different geographical origins and processing conditions (42). The establishment of such comprehensive databases of oil-specific biomarkers is essential for regulatory agencies and industry stakeholders to maintain consumer trust and ensure food safety (43).

### 3.4 Correlation analysis of volatile and non-volatile metabolites

**Figure 5:**
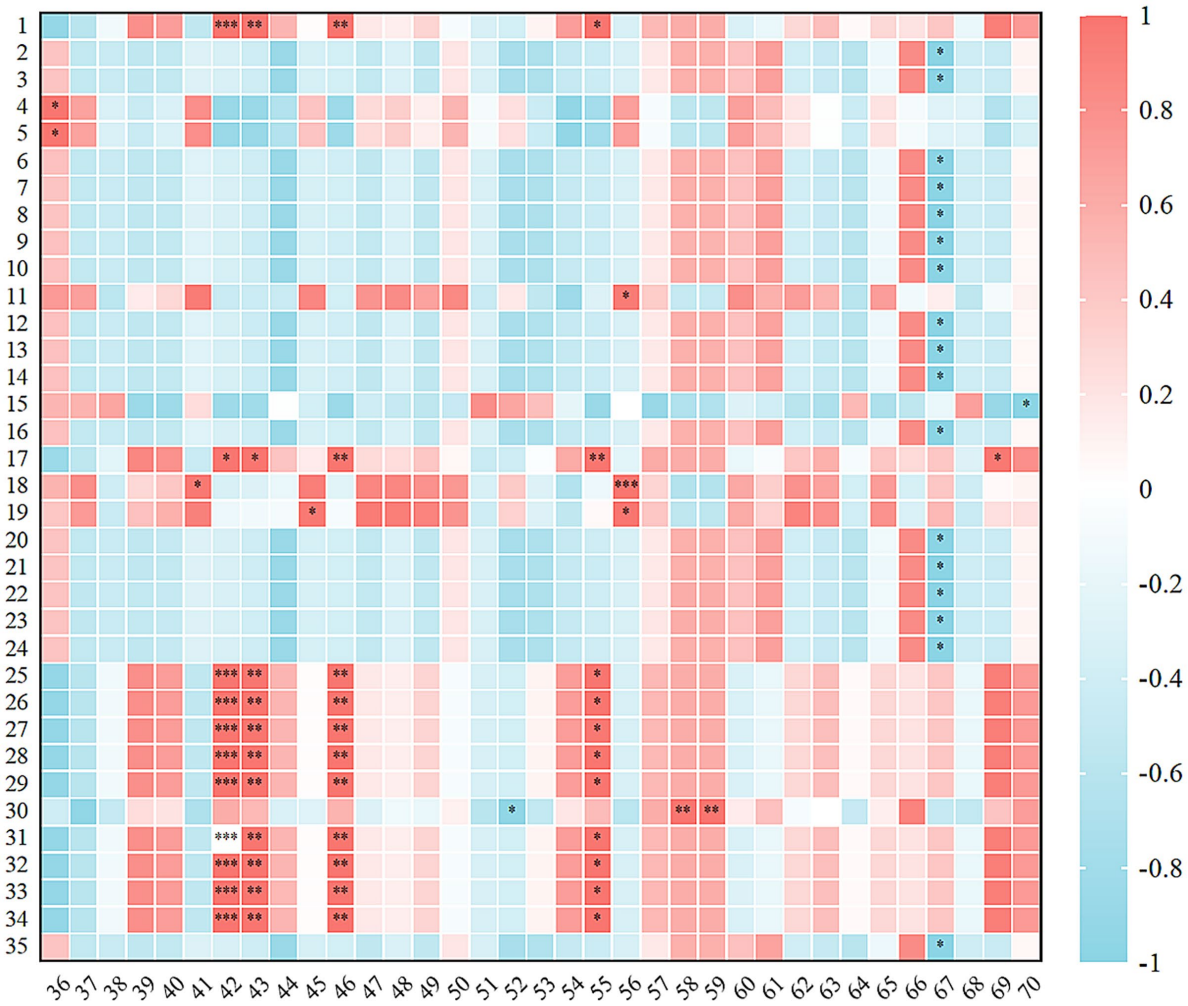
Correlation heatmap of Pearson correlation coefficients between key volatile differential compounds (ROAV > 1) and non-volatile differential compounds (TOP 15) in walnut (WO), olive (OO), flaxseed (FSO), and camellia (CO) oils. Red and blue colors represent positive and negative correlations, respectively. Asterisks (*) denote significant correlations (*p <* 0.05).

Pearson correlation analysis was performed to explore the associations between volatile and non-volatile compounds identified in the four vegetable oils (Figure 5). The correlation matrix revealed that significant positive correlations were primarily concentrated among non-volatile metabolites themselves, particularly within clusters of structurally related compounds (e.g., cyclic peptides, saponins, and glycosides), whereas correlations involving volatiles were more sporadic and less clustered. Notably, several robust positive correlations emerged between specific non-volatile metabolites and volatile alcohols or aldehydes, suggesting functional cross-talk between secondary metabolic pathways and core lipid metabolism. For instance, the strong positive correlation observed between glycosides/saponins (Compounds 1, 26, 27) and medium-chain fatty alcohols (e.g., 3-octanol and 1-nonanol, Compounds 42, 43, 46) can be rationalized by the hydrolysis of glycosidic sugar moieties, which releases monosaccharides that enter glycolysis and generate acetyl-CoA. This acetyl-CoA pool serves as a precursor for the synthesis of medium-chain fatty acids, which are subsequently reduced to their corresponding alcohols by alcohol dehydrogenases (44). In a related vein, a cluster of cyclic peptides, macrocyclic compounds, and phenolic lipids (Compounds 25, 28, 32, 33, 34) also exhibited positive correlation with 3-octanol (Compound 42). This is plausibly attributed to their collective modulation of fatty acid metabolism—through precursor provision (saponins/glycosides), enzyme activity regulation (cyclic peptides), and inhibition of fatty acid *β*-oxidation (macrocyclic/phenolic lipids), all of which converge to enhance the accumulation of the volatile alcohol. Beyond sugar-driven mechanisms, a distinct positive correlation was found between the alkaloid (S)-autumnaline (Compound 18) and the unsaturated aldehyde (E)-2-octenal (Compound 56). This linkage is likely mediated by the oxidative by-products of alkaloid biosynthesis; the conversion of tyrosine to the benzyltetrahydroisoquinoline skeleton may generate hydrogen peroxide, which in turn activates lipoxygenase (LOX)—a key enzyme in the oxidation of linoleic acid to C8 volatile aldehydes (45). Thus, enhanced alkaloid synthesis can indirectly promote LOX-catalyzed lipid oxidation, leading to the coupled accumulation of both metabolites (46). Collectively, these correlations underscore the intricate interplay between primary lipid metabolism and secondary metabolite pathways in vegetable oils. The findings not only highlight potential regulatory nodes (e.g., fatty acid synthase, lipoxygenase, and glycosyltransferases) that govern the concurrent biosynthesis of flavor-active volatiles and nutritionally relevant non-volatiles, but also provide a metabolomic basis for understanding how intrinsic compositional traits shape the overall quality and sensory profile of each oil type. Future investigations may focus on validating these regulatory interactions through targeted enzyme assays or genetic approaches, and on exploring how processing conditions might modulate these correlations to optimize oil quality.

## 4 Conclusion

This study presents an integrated metabolomic framework for the comprehensive characterization of four high-value vegetable oils—camellia, flaxseed, olive, and walnut—by jointly profiling volatile and non-volatile metabolites. The principal novelty resides not in the individual enumeration of compounds, but in the functional cross-talk revealed between these two chemical dimensions, which provides mechanistic insights into oil quality that are unattainable through single-platform analyses. The integrated approach uncovers previously unrecognized biochemical linkages that govern flavor formation. Correlation analysis demonstrates a significant positive association between glycosides/saponins and medium-chain fatty alcohols, suggesting that glycoside hydrolysis supplies monosaccharides for glycolytic acetyl-CoA production, thereby driving the biosynthesis of medium-chain fatty acids and their corresponding alcohols. Concurrently, the covariation of an alkaloid ((S)-autumnaline) with an unsaturated aldehyde ((E)-2-octenal) implies that secondary metabolites may indirectly modulate lipoxygenase activity, influencing lipid oxidation pathways. These findings offer a mechanistic explanation for the distinct flavor profiles observed among oils with comparable fatty acid compositions. From a practical standpoint, this work establishes a dual-marker authentication strategy that integrates both volatile and non-volatile biomarkers. While non-volatile markers—such as secoiridoids for olive oil and ecdysteroids for camellia oil—provide high species specificity, their discriminatory power is substantially reinforced by complementary volatile markers, including 1-penten-3-one for olive oil and hexanal for camellia oil. This synergistic system, validated through PLS-DA modelling, is more resilient against sophisticated adulteration tactics that may target single chemical classes. The distinct phospholipid signatures in walnut oil and steroidal alkaloids in flaxseed oil, when considered alongside their unique volatile profiles, constitute a robust chemical barcode for each oil type. Collectively, these findings advance the understanding of vegetable oil quality by elucidating the metabolic interactions that connect primary and secondary metabolism to sensory properties and oxidative stability. The integrated biomarker panel and the underlying mechanistic framework offer a scientifically rigorous foundation for authenticity verification, quality control, and product optimization. Future investigations incorporating broader sample sets and multi-omics approaches—including transcriptomics and proteomics—will further elucidate the regulatory networks governing these metabolic pathways, facilitating the translation of plant lipid metabolomics from fundamental research to industrial application.

## Data Availability

The original contributions presented in the study are included in the article/Supplementary material, further inquiries can be directed to the corresponding author/s.

## Footnotes

1
https://www.hmdb.ca

2
https://www.massbank.jp/

3
https://www.lipidmaps.org

4
https://www.mzcloud.org

5
https://www.omicshare.com/tools

## Supplementary material

SUPPLEMENTARY FIGURE S1 Total ion current (TIC) chromatograms of volatile compounds in four vegetable oils detected by HS-SPME-GC-MS. **(A)** Camellia oil; **(B)** Flaxseed oil; **(C)** Olive oil; **(D)** Walnut oil.

SUPPLEMENTARY FIGURE S2 TIC chromatograms of four oil samples analyzed by LC-MS. **(A1–D1)** Negative ion mode (NEG); **(A2–D2)** Positive ion mode (POS). **(A)** Camellia oil; **(B)** Flaxseed oil; **(C)** Olive oil; **(D)** Walnut oil.

SUPPLEMENTARY FIGURE S3 Permutation test plots of the six pairwise PLS-DA models for the four vegetable oils. **(A)** FSO vs CO; **(B)** OO vs CO; **(C)** OO vs FSO; **(D)** OO vs WO; **(E)** WO vs CO; **(F)** WO vs FSO.
