# Supplementary material for: Comparative metabolomics of high-unsaturated fatty acid oils: exploring volatile and nonvolatile metabolite differences in olive, walnut, flaxseed, and camellia oils
Source: Front Nutr. 2026 Aug 3;13:1853929. doi: 10.3389/fnut.2026.1853929 (PMC13478218; doi:10.3389/fnut.2026.1853929)
Supplement: Supplementary file 1 [file Table_1.docx]

Table S1. Retention Time and Retention Index Table for Alkane Standards

| Alkanes | RT(min) | RI |
| --- | --- | --- |
| C9 | 2.66247 | 900.00 |
| C10 | 4.28381 | 1000.0 |
| C11 | 6.92681 | 1100.0 |
| C12 | 9.92963 | 1200.0 |
| C13 | 12.8614 | 1300.0 |
| C14 | 15.6065 | 1400.0 |
| C15 | 18.174 | 1500.0 |
| C16 | 20.5994 | 1600.0 |
| C17 | 22.9048 | 1700.0 |
| C18 | 25.0903 | 1800.0 |
| C19 | 27.178 | 1900.0 |
| C20 | 29.1814 | 2000.0 |
| C21 | 31.1048 | 2100.0 |
| C22 | 32.9527 | 2200.0 |
| C23 | 33.8677 | 2300.0 |
