# Supplementary material for: Comparative metabolomics of high-unsaturated fatty acid oils: exploring volatile and nonvolatile metabolite differences in olive, walnut, flaxseed, and camellia oils
Source: Front Nutr. 2026 Aug 3;13:1853929. doi: 10.3389/fnut.2026.1853929 (PMC13478218; doi:10.3389/fnut.2026.1853929)
Supplement: Supplementary file 2 [file Table_2.docx]

Table S2. Statistics of Up/Down-Regulated Differential Metabolites

| Group | Cpd_all | Cpd_diff | Cpd_diff_up | Cpd_diff_down |
| --- | --- | --- | --- | --- |
| WO vs FSO | 1186 | 654 | 182 | 472 |
| OO vs WO | 1186 | 692 | 350 | 342 |
| FSO vs CO | 1186 | 449 | 113 | 336 |
| OO vs CO | 1186 | 548 | 101 | 447 |
| OO vs FSO | 1186 | 712 | 267 | 445 |

Notes:Cpd_all: Total number of compounds detected; Cpd_diff: Number of differential metabolites in the comparison; Cpd_diff_up: Up-regulated metabolites; Cpd_diff_down: Down-regulated metabolites.
