# Supplementary material for: Comparative metabolomics of high-unsaturated fatty acid oils: exploring volatile and nonvolatile metabolite differences in olive, walnut, flaxseed, and camellia oils
Source: Front Nutr. 2026 Aug 3;13:1853929. doi: 10.3389/fnut.2026.1853929 (PMC13478218; doi:10.3389/fnut.2026.1853929)
Supplement: Supplementary file 3 [file Table_3.docx]

Table S3. Compound-Code Cross-Reference for Figure 5

|  | Compound |
| --- | --- |
| 1 | Solasodine base + 2H, O-Hex-Hex-Hex-Pen |
| 2 | (3S,6R,12S)-6-benzyl-3-isobutyl-9-[6-(oxiran-2-yl)-6-oxo-hexyl]-1,4,7,10-tetrazabicyclo[10.4.0]hexadecane-2,5,8,11-tetrone |
| 3 | Anthranoyllycoctonine |
| 4 | 2,4-Dihydroxybutanoic acid |
| 5 | 2-Ketobutyric acid |
| 6 | Flurandrenolide |
| 7 | Denticulaflavonol |
| 8 | LPA(20:5) |
| 9 | COSTUNOLIDE |
| 10 | Goniothalenol |
| 11 | Liquidambaric acid |
| 12 | (5S,9R)-14-(hydroxymethyl)-5,9-dimethyl-tetracyclo[11.2.1.01,10.04,9]hexadec-14-ene-5-carboxylic acid |
| 13 | 4-[2-[(1R,4aS,5R,8aS)-5-(hydroxymethyl)-5,8a-dimethyl-2-methylene-decalin-1-yl]ethyl]-2H-furan-5-one |
| 14 | (8E)-10-hydroxy-5,9,12,13,16-pentamethyl-4-oxatricyclo[10.3.1.03,5]hexadeca-1(15),8-dien-2-one |
| 15 | N-Linoleoyl Lysine |
| 16 | paulownin |
| 17 | PI(18:1/18:1) |
| 18 | (S)-Autumnaline |
| 19 | Debromoaplysiatoxin |
| 20 | 17-[2,3-dihydroxy-6-methyl-6-[3,4,5-trihydroxy-6-(hydroxymethyl)oxan-2-yl]oxyheptan-2-yl]-2,3,14-trihydroxy-10,13-dimethyl-2,3,4,5,9,11,12,15,16,17-decahydro-1H-cyclopenta[a]phenanthren-6-one |
| 21 | N-arachidonoyl-glutamate |
| 22 | Bufalin |
| 23 | [(2E,6E)-8-(2,6-dihydroxy-4-methyl-phenyl)-2,6-dimethyl-1-(3-methylbut-2-enyl)octa-2,6-dienyl] acetate |
| 24 | 12-HEPE |
| 25 | Cyclolinopeptide_I |
| 26 | Zingiberensis saponin I |
| 27 | Tomatoside_A |
| 28 | 24,27-dibenzyl-12,21-diisobutyl-9-isopropyl-15,18-disec-butyl-1,7,10,13,16,19,22,25,28-nonazatricyclo[28.3.0.03,7]tritriacontane-2,8,11,14,17,20,23,26,29-nonone |
| 29 | Ampeloside_Bf1 |
| 30 | 1-18:3-2-18:3-phosphatidylinositol |
| 31 | Melongoside_P |
| 32 | Cyclolinopeptide_H |
| 33 | 5-nonadecylbenzene-1,3-diol |
| 34 | FR900359 |
| 35 | 5,6,16-Trihydroxygrayanotox-10-en-3-yl_hexopyranoside |
| 36 | Ethanol |
| 37 | 1-Hexanol |
| 38 | 3-Hexen-1-ol, (Z)- |
| 39 | 1-Pentanol |
| 40 | 1-Octen-3-ol |
| 41 | 1-Heptanol |
| 42 | 3-Octanol |
| 43 | 1-Nonanol |
| 44 | Benzyl alcohol |
| 45 | Linalool |
| 46 | 2-Heptanol |
| 47 | p-Xylene |
| 48 | Hexanal |
| 49 | Nonanal |
| 50 | Pentanal |
| 51 | Benzeneacetaldehyde |
| 52 | Benzaldehyde |
| 53 | Butanal, 2-methyl- |
| 54 | 2-Pentenal, (E)- |
| 55 | 2,6-Nonadienal, (E,E)- |
| 56 | 2-Octenal, (E)- |
| 57 | Butanoic acid |
| 58 | Hexanoic acid |
| 59 | Hexanoic acid |
| 60 | Acetic acid |
| 61 | Propanoic acid, 2-methyl- |
| 62 | Butanoic acid, 3-methyl- |
| 63 | Butanoic acid, 2-methyl- |
| 64 | α-Phellandrene |
| 65 | β-Myrcene |
| 66 | 2-Heptanone |
| 67 | 2-Octanone |
| 68 | 1-Penten-3-one |
| 69 | Furan, 2-pentyl- |
| 70 | Hexanoic acid, ethyl ester |
