# Supplementary material for: Comparative metabolomics of high-unsaturated fatty acid oils: exploring volatile and nonvolatile metabolite differences in olive, walnut, flaxseed, and camellia oils
Source: Front Nutr. 2026 Aug 3;13:1853929. doi: 10.3389/fnut.2026.1853929 (PMC13478218; doi:10.3389/fnut.2026.1853929)
Supplement: Supplementary file 4 [file Table_4.docx]

| Table S4 The volatile compounds of four types of highly unsaturated fatty acid vegetables oils | | | | | | | | | | | | |
| --- | --- | --- | --- | --- | --- | --- | --- | --- | --- | --- | --- | --- |
| Compounds | RI | Formula | CAS | Content/mg/mL | | | | Threshold/ppb | ROAV | | | |
|  |  |  |  | WO | OO | FSO | CO |  | WO | OO | FSO | CO |
| **Alcohols** |  |  |  |  |  |  |  |  |  |  |  |  |
| Ethanol | 927.5 | C_2_H_6_O | 64-17-5 | 5.297±0.142^a^ | 2.979±0.350^b^ | ND | 4.864±0.095^a^ | 8 | 100 | 42.617 | ＜0.1 | 100 |
| 1-Hexanol | 1,349.2 | C_6_H_14_O | 111-27-3 | 0.581±0.022^a^ | 0.369±0.023^b^ | ND | 0.031±0.010^c^ | 10 | 8.774 | 5.99 | ＜0.1 | 0.516 |
| 3-Hexen-1-ol, (Z)- | 1,376.6 | C_6_H_12_O | 928-96-1 | 0.004±0.001^b^ | 0.134±0.041^a^ | 0.033±0.001^b^ | ND | 70 | ＜0.1 | 0.311 | 0.289 | ＜0.1 |
| 1-Pentanol | 1,247.1 | C_5_H_12_O | 71-41-0 | 0.271±0.007^b^ | 0.031±0.006^c^ | 0.416±0.039^a^ | 0.042±0.004^c^ | 120 | 0.341 | ＜0.1 | 2.097 | ＜0.1 |
| 1-Octanol | 1,553.9 | C_8_H_18_O | 111-87-5 | 0.015±0.001^a^ | 0.005±0.002^b^ | ND | 0.004±0.001^b^ | 42 | ＜0.1 | ＜0.1 | ＜0.1 | ＜0.1 |
| Benzeneethanol, 4-hydroxy- | 3,541.0 | C_8_H_10_O_2_ | 501-94-0 | 0.004±0.001^b^ | 0.033±0.005^a^ | ND | ND |  |  |  |  |  |
| 1-Octen-3-ol | 1,445.6 | C_8_H_16_O | 3391-86-4 | 0.088±0.007^b^ | 0.006±0.002^d^ | 0.112±0.005^a^ | 0.016±0.003^c^ | 1 | 13.277 | 1.009 | 67.934 | 2.554 |
| 1-Penten-3-ol | 1,157.5 | C_5_H_10_O | 616-25-1 | 0.039±0.006^c^ | 0.206±0.061^b^ | 0.970±0.111^a^ | 0.049±0.003^c^ | 400 | ＜0.1 | ＜0.1 | 1.466 | ＜0.1 |
| 2-Propanol, 1-methoxy- | 1,124.4 | C_4_H_10_O_2_ | 107-98-2 | 0.050±0.008^b^ | 0.021±0.006^c^ | 0.102±0.015^a^ | 0.017±0.003^c^ | 10000 | ＜0.1 | ＜0.1 | ＜0.1 | ＜0.1 |
| 1-Butanol | 1,143.9 | C_4_H_10_O | 71-36-3 | 0.020±0.001^b^ | 0.002±0.001^d^ | 0.056±0.004^a^ | 0.008±0.002^c^ | 500 | ＜0.1 | ＜0.1 | ＜0.1 | ＜0.1 |
| Phenylethyl Alcohol | 1,894.7 | C_8_H_10_O | 60-12-8 | 0.007±0.001b^c^ | 0.021±0.007^a^ | 0.013±0.001^b^ | 0.001±0.001^c^ | 1000 | ＜0.1 | ＜0.1 | ＜0.1 | ＜0.1 |
| 1-Heptanol | 1,451.2 | C_7_H_16_O | 111-70-6 | 0.024±0.002^a^ | 0.006±0.002^b^ | ND | 0.004±0.001^b^ | 3 | 1.231 | 0.348 | ＜0.1 | 0.234 |
| 1-Hexanol, 2-ethyl- | 1,485.5 | C_8_H_18_O | 104-76-7 | 0.044±0.003^a^ | ND | 0.033±0.005^b^ | 0.037±0.007^ab^ |  |  |  |  |  |
| 2-Furanmethanol, 5-ethenyltetrahydro-α,α,5-trimethyl-, cis- | 1,434.5 | C_10_H_18_O_2_ | 5989-33-3 | ND | ND | 0.006±0.001 | ND | 320 | ＜0.1 | ＜0.1 | ＜0.1 | ＜0.1 |

Table S4 (continued)

| Compounds | RI | Formula | CAS | Content/mg/mL | | | | Threshold/ppb | ROAV | | | |
| --- | --- | --- | --- | --- | --- | --- | --- | --- | --- | --- | --- | --- |
|  |  |  |  | WO | OO | FSO | CO |  | WO | OO | FSO | CO |
| 3-Octanol | 1,391.2 | C_8_H_18_O | 589-98-0 | ND | ND | 0.0154±0.001 | ND | 18 | ＜0.1 | ＜0.1 | 0.518 | ＜0.1 |
| 2-Hexen-1-ol, (E)- | 1,398.9 | C6H12O | 928-95-0 | 0.068±0.002b | 0.855±0.191^a^ | 0.115±0.016^b^ | ND |  |  |  |  |  |
| 2-Penten-1-ol, (Z)- | 1,313.6 | C_5_H_10_O | 1576-95-0 | ND | 0.155±0.049^a^ | 0.091±0.003^b^ | ND |  |  |  |  |  |
| 1-Nonanol | 1,656.3 | C_9_H_20_O | 143-08-8 | 0.001±0.001^b^ | 0.001±0^b^ | 0.012±0.001^a^ | ND | 50 | ＜0.1 | ＜0.1 | 0.145 | ＜0.1 |
| 2,3-Butanediol | 1,569.9 | C_4_H_10_O_2_ | 513-85-9 | 0.039±0.004^a^ | 0.0001±0^b^ | 0.041±0.003^a^ | 0.002±0.001^b^ |  |  |  |  |  |
| 1-Hydroxy-2-butanone | 1,355.8 | C_4_H_8_O_2_ | 5077-67-8 | 0.014±0^b^ | ND | 0.280±0.018^a^ | 0.012±0.004^b^ |  |  |  |  |  |
| Benzyl alcohol | 1,859.5 | C_7_H_8_O | 100-51-6 | 0.003±0^b^ | 0.005±0.002^a^ | 0.005±0^a^ | 0.001±0^c^ | 1.2 | 0.345 | 0.686 | 2.636 | 0.111 |
| 2-Penten-1-ol, (E)- | 1,305.3 | C_5_H_10_O | 1576-96-1 | ND | 0.031±0.009^b^ | 0.101±0.003^a^ | ND |  |  |  |  |  |
| Linalool | 1,542.7 | C_10_H_18_O | 78-70-6 | 0.012±0.001^a^ | ND | 0.005±0.002^b^ | 0.002±0.001^c^ | 4 | 0.442 | 0.269 | 0.691 | ＜0.1 |
| cis-Hept-4-enol | 1,488.9 | C_7_H_14_O | 6191-71-5 | ND | ND | 0.004±0 | ND |  |  |  |  |  |
| 1,4-Butanediol | 1,913.2 | C_4_H_10_O_2_ | 110-63-4 | 0.004±0^a^ | 0.003±0.001^b^ | 0.003±0^b^ | 0.003±0^b^ |  |  |  |  |  |
| 2-Heptanol | 1,318.1 | C_7_H_16_O | 543-49-7 | 0.008±0.001^b^ | ND | 0.083±0.002^a^ | ND | 41 | ＜0.1 | ＜0.1 | 1.222 | ＜0.1 |
| 2-Pentanol, 4-methyl- | 1,220.4 | C_6_H_14_O | 108-11-2 | ND | ND | 0.018±0.001 | ND |  |  |  |  |  |
| 2-Butanol | 1,024.2 | C_4_H_10_O | 78-92-2 | 0.007±0.006^b^ | ND | 0.145±0.017^a^ | ND | 43000 | ＜0.1 | ＜0.1 | ＜0.1 | ＜0.1 |
| 1-Propanol, 2-methyl- | 1,095.8 | C_4_H_10_O | 78-83-1 | 0.007±0.003^a^ | 0.003±0.003^ab^ | 0.007±0.001^a^ | 0.001±0.001^b^ | 360 | ＜0.1 | ＜0.1 | ＜0.1 | ＜0.1 |
| 1,5-Hexadien-3-ol | 1,992.5 | C_6_H_10_O | 924-41-4 | 0.008±0.001 | ND | ND | ND |  |  |  |  |  |
| 3-Nonen-1-ol, (Z)- | 1,677.5 | C_9_H_18_O | 10340-23-5 | ND | 0.001±0 | ND | ND |  |  |  |  |  |

Table S4 (continued)

| Compounds | RI | Formula | CAS | Content/mg/mL | | | | Threshold/ppb | ROAV | | | |
| --- | --- | --- | --- | --- | --- | --- | --- | --- | --- | --- | --- | --- |
|  |  |  |  | WO | OO | FSO | CO |  | WO | OO | FSO | CO |
| 2-Hepten-1-ol, (E)- | 1,504.0 |  |  | ND | ND | 0.017±0.001 | ND |  |  |  |  |  |
| 3-Hexen-1-ol | 1,357.3 | C_6_H_12_O | 544-12-7 | ND | 0.008±0.002 | 0.008±0 | ND |  |  |  |  |  |
| 4-Heptanol | 1,652.3 | C7H16O | 589-55-9 | ND | ND | 0.002±0 | ND |  |  |  |  |  |
| 3-Pentanol | 1,111.0 | C_5_H_12_O | 584-02-1 | ND | ND | 0.085±0.008 | ND |  |  |  |  |  |
| 1,2-Butanediol | 1,551.3 | C_4_H_10_O_2_ | 584-03-2 | ND | ND | 0.003±0 | ND |  |  |  |  |  |
| 2-Hexen-1-ol, (Z)- | 1,408.2 | C_6_H_12_O | 928-94-9 | ND | 0.001±0 | ND | ND |  |  |  |  |  |
| 2-Nonen-1-ol, (Z)- | 1,707.9 | C_9_H_18_O | 41453-56-9 | ND | ND | 0.002±0 | ND |  |  |  |  |  |
| 1-Butanol, 3-methyl- | 1,204.7 | C_5_H_12_O | 123-51-3 | 0.008±0.001^b^ | ND | 0.016±0.001^a^ | ND |  |  |  |  |  |
| 5-Hepten-2-ol, 6-methyl- | 1,459.2 | C_8_H_16_O | 1569-60-4 | 0.003±0^b^ | ND | 0.029±0.002^a^ | ND | 2000 | ＜0.1 | ＜0.1 | ＜0.1 | ＜0.1 |
| 2H-Pyran-2-methanol, tetrahydro- | 1,952.1 | C_6_H_12_O_2_ | 100-72-1 | 0.006±0.002 | 0.013±0.012 | 0.01±0 |  |  |  |  |  |  |
| 4-Heptanol | 1,652.3 | C_7_H_16_O | 589-55-9 | ND | ND | 0.002±0 |  |  |  |  |  |  |
| SUM |  |  |  | 6.663 | 4.901 | 2.841 |  |  |  |  |  |  |
| **Aromatic hydrocarbons** | | | | | | | | | | | | |
| Naphthalene | 1713.8 | C_10_H_8_ | 91-20-3 | 0.002 ±0^a^ | 0.001 ±0^b^ | 0.002 ±0^a^ | 0.002 ±0.001^a^ | 300 | ＜0.1 | ＜0.1 | ＜0.1 | ＜0.1 |
| o-Cymene | 1351.06 | C_10_H_14_ | 527-84-4 | 0.015 ±0.012 | 0.013 ±0.005 | 0.018 ±0.016 | 0.025 ±0.004 |  |  |  |  |  |
| Benzene, 1,2,4,5-tetramethyl- | 1416.39 | C_10_H_14_ | 95-93-2 | 0.076 ±0.009 | 0.055 ±0.018 | 0.058 ±0.051 | 0.064 ±0.013 |  |  |  |  |  |

Table S4 (continued)

| Compounds | RI | Formula | CAS | Content/mg/mL | | | | Threshold/ppb | ROAV | | | |
| --- | --- | --- | --- | --- | --- | --- | --- | --- | --- | --- | --- | --- |
|  |  |  |  | WO | OO | FSO | CO |  | WO | OO | FSO | CO |
| Benzene, 1,2,3,4-tetramethyl- | 1468.25 | C_10_H_14_ | 488-23-3 | 0.029 ±0.003 | 0.020 ±0.006 | 0.022 ±0.019 | 0.025 ±0.005 |  |  |  |  |  |
| p-Xylene | 1121.94 | C_8_H_10_ | 106-42-3 | 0.064 ±0.005^a^ | 0.019 ±0.007^c^ | 0.041 ±0.005^b^ | 0.019 ±0.007^c^ | 50 | 0.193 | ＜0.1 | 0.49 | ＜0.1 |
| Naphthalene, 2-methyl- | 1825.89 | C_11_H_10_ | 91-57-6 | ND | ND | 0.001 ±0 | ND | 10 | ＜0.1 | ＜0.1 | ＜0.1 | ＜0.1 |
| Benzene, (2-methyl-1-propenyl)- | 1454.42 | C_10_H_12_ | 768-49-0 | 0.009 ±0.001^ab^ | 0.007 ±0.002^b^ | 0.011 ±0.001^a^ | 0.009 ±0.002^ab^ |  |  |  |  |  |
| p-Cymene | 1255.02 | C_10_H_14_ | 99-87-6 | 0.031 ±0.016^a^ | 0.021 ±0.01^ab^ | 0.010 ±0.004^b^ | 0.008 ±0.007^b^ |  |  |  |  |  |
| Toluene | 1026.10 | C_7_H_8_ | 108-88-3 | 0.027 ±0^b^ | 0.026 ±0.008^b^ | 0.046 ±0.008^a^ | 0.029 ±0.004^b^ | 2140 | ＜0.1 | ＜0.1 | ＜0.1 | ＜0.1 |
| SUM |  |  |  | 0.253 | 0.163 | 0.207 | 0.180 |  |  |  |  |  |
| **Phenols** |  |  |  |  |  |  |  |  |  |  |  |  |
| 2,6-Bis(1,1-dimethylethyl)-4-(1-oxopropyl)phenol | 1917.76 | C_17_H_26_O_2_ | 14035-34-8 | 0.007 ±0^a^ | 0.005±0.001^ab^ | 0.003 ±0.001^b^ | 0.004 ±0.003^b^ |  |  |  |  |  |
| 2-Methoxy-5-methylphenol | 1935.93 | C_8_H_10_O_2_ | 1195-09-1 | 0.001 ±0 | ND | ND | ND |  |  |  |  |  |
| SUM |  |  |  | 0.008 | 0.005 | 0.003 | 0.004 |  |  |  |  |  |
| **Ethers** |  |  |  |  |  |  |  |  |  |  |  |  |
| Anethole | 1808.17 |  |  | 0.001 ±0 | ND | ND | ND |  |  |  |  |  |
| Estragole | 1652.6 | C_10_H_12_O | 140-67-0 | 0.003 ±0 | ND | ND | ND |  |  |  |  |  |
| SUM |  |  |  | 0.004 | 0 | 0 | 0 |  |  |  |  |  |
| **Aldehydes** |  |  |  |  |  |  |  |  |  |  |  |  |
| Hexanal | 1069.94 | C_6_H_12_O | 66-25-1 | 1.085 ±0.037^a^ | 0.194 ±0.059^d^ | 0.662 ±0.067^b^ | 0.414 ±0.021^c^ | 4 | 40.976 | 7.875 | 100 | 17.03 |

Table S4 (continued)

| Compounds | RI | Formula | CAS | Content/mg/mL | | | | Threshold/ppb | ROAV | | | |
| --- | --- | --- | --- | --- | --- | --- | --- | --- | --- | --- | --- | --- |
|  |  |  |  | WO | OO | FSO | CO |  | WO | OO | FSO | CO |
| 2-Pentenal, 2-methyl- | 1134.63 | C_6_H_10_O | 623-36-9 | ND | 0.003 ±0.001^b^ | 0.013 ±0.001^a^ | ND |  |  |  |  |  |
| 2-Butenal | 1027.18 |  |  | ND | ND | 0.004 ±0.001 | 0.001 ±0 |  |  |  |  |  |
| 2-Hexenal | 1203.93 |  |  | 0.026 ±0.003^b^ | ND | 0.069 ±0.022^a^ | 0.004 ±0.002^c^ |  |  |  |  |  |
| Benzaldehyde, 2,4-dimethyl- | 1789.47 | C_9_H_10_O | 15764-16-6 | 0.005 ±0 | 0.003 ±0.001 | 0.005 ±0.001 | 0.004 ±0.001 |  |  |  |  |  |
| Butanal, 3-methyl- | 909.56 | C_5_H_10_O | 590-86-3 | 0.005 ±0.001^b^ | 0.011 ±0.003^ab^ | 0.011 ±0.01^a^ | ND |  |  |  |  |  |
| Nonanal | 1382.8 | C_9_H_18_O | 124-19-6 | 0.033 ±0.002^a^ | 0.010 ±0.006^b^ | 0.025 ±0.001^a^ | 0.011 ±0.002^b^ | 1 | 5.042 | 1.67 | 15.108 | 1.736 |
| 4-Oxohex-2-enal | 1577.3 | C_6_H_8_O_2_ | 20697-55-6 | ND | 0.025 ±0.011 | ND | ND |  |  |  |  |  |
| Pentanal | 965.51 | C_5_H_10_O | 110-62-3 | 0.154 ±0.003^a^ | 0.039 ±0.013^d^ | 0.095 ±0.013^c^ | 0.114 ±0.002^b^ | 12 | 1.944 | 0.526 | 4.778 | 1.569 |
| 3-Hexenal, (Z)- | 1125.45 | C_6_H_10_O | 6789-80-6 | ND | ND | ND | ND | 0.25 | ＜0.1 | ＜0.1 | ＜0.1 | ＜0.1 |
| Octanal | 1276.85 | C_8_H_16_O | 124-13-0 | ND | ND | ND | ND | 1.4 | ＜0.1 | ＜0.1 | ＜0.1 | ＜0.1 |
| 2,4-Hexadienal, (E,E)- | 1385.56 | C_6_H_8_O | 142-83-6 | 0.001 ±0^b^ | 0.002 ±0.001^a^ | 0.001 ±0^b^ | ND | 60 | ＜0.1 | ＜0.1 | ＜0.1 | ＜0.1 |
| 2,4-Heptadienal, (E,E)- | 1449.52 | C_7_H_10_O | 4313-03-5 | 0.004 ±0^b^ | 0.005 ±0.002^b^ | ND | 0.011 ±0.002^a^ |  |  |  |  |  |
| Benzeneacetaldehyde | 1618.83 | C_8_H_8_O | 122-78-1 | ND | 0.003 ±0 | ND | ND | 4 | ＜0.1 | 0.127 | ＜0.1 | ＜0.1 |
| Benzaldehyde | 1500.56 | C_7_H_6_O | 100-52-7 | 0.049 ±0.011 | 0.059 ±0.052 | 0.027 ±0.006 | 0.016 ±0.003 | 3 | 2.452 | 1.759 | 5.374 | 0.861 |
| Butanal, 2-methyl- | 906.31 | C_5_H_10_O | 96-17-3 | 0.002±0.003^b^ | 0.008 ±0.003^a^ | 0.004 ±0.003^b^ | ND | 1 | 0.292 | 1.222 | 2.383 | ＜0.1 |
| 2-Pentenal, (E)- | 1115.96 | C_5_H_8_O | 1576-87-0 | ND | 0.007 ±0.002^a^ | 0.009 ±0.002^a^ | 0.001 ±0^b^ | 1.5 | ＜0.1 | 0.759 | 1.485 | 0.129 |
| 2,6-Nonadienal, (E,E)- | 1571.9 | C_9_H_14_O | 17587-33-6 | 0.001 ±0^b^ | ND | 0.005 ±0.001^a^ | ND | 0.09 | 1.199 | ＜0.1 | 33.34 | ＜0.1 |
| 2-Octenal, (E)- | 1415.45 | C_8_H_14_O | 2548-87-0 | 0.003 ±0 | ND | ND | ND | 3 | 0.157 | ＜0.1 | ＜0.1 | ＜0.1 |
| SUM |  |  |  | 1.369 | 0.371 | 0.928 | 0.576 |  |  |  |  |  |
| **Alkynes** |  |  |  |  |  |  |  |  |  |  |  |  |
| 3-Decyne | 1020.7 | C_10_H_18_ | 2384-85-2 | ND | 0.003±0.001 | ND | ND |  |  |  |  |  |

Table S4 (continued)

| Compounds | RI | Formula | CAS | Content/mg/mL | | | | Threshold/ppb | ROAV | | | |
| --- | --- | --- | --- | --- | --- | --- | --- | --- | --- | --- | --- | --- |
|  |  |  |  | WO | OO | FSO | CO |  | WO | OO | FSO | CO |
| SUM |  |  |  | 0 | 0.003 | 0 | 0 |  |  |  |  |  |
| **Acids** |  |  |  |  |  |  |  |  |  |  |  |  |
| Propanoic acid | 1524.15 | C_3_H_6_O_2_ | 79-09-4 | 0.490 ±0.055^a^ | 0.239 ±0.077^b^ | 0.386 ±0.027^a^ | 0.465 ±0.045^a^ | 5000 | ＜0.1 | ＜0.1 | ＜0.1 | ＜0.1 |
| Butanoic acid | 1615.96 | C_4_H_8_O_2_ | 107-92-6 | 0.169 ±0.008^a^ | 0.080 ±0.027^b^ | 0.183 ±0.021^a^ | 0.160 ±0.026^a^ | 240 | 0.107 | ＜0.1 | 0.461 | 0.11 |
| Hexanoic acid | 1834.51 | C_6_H_12_O_2_ | 142-62-1 | ND | 0.031 ±0.011^b^ | 0.336 ±0.061^a^ | 0.333 ±0.018^a^ | 93 | ＜0.1 | ＜0.1 | 2.185 | 0.588 |
| Acetic acid | 1436.37 | C_2_H_4_O_2_ | 64-19-7 | 0.378 ±0.043^a^ | 0.127 ±0.043^c^ | 0.213 ±0.013^b^ | 0.346 ±0.006^a^ | 210 | 0.272 | ＜0.1 | 0.614 | 0.271 |
| Octanoic acid | 2051.4 | C_8_H_16_O_2_ | 124-07-2 | 0.026 ±0.002^a^ | 0.005 ±0.001^c^ | 0.015 ±0.002^b^ | 0.007 ±0.001^c^ | 910 | ＜0.1 | ＜0.1 | ＜0.1 | ＜0.1 |
| Propanoic acid, 2-methyl- | 1558.05 | C_4_H_8_O_2_ | 79-31-2 | 0.014 ±0.003 | 0.007 ±0.002 | 0.011 ±0.009 | 0.016 ±0.002 | 10 | 0.214 | 0.113 | 0.657 | 0.266 |
| Butanoic acid, 3-methyl- | 1659.42 | C_5_H_10_O_2_ | 503-74-2 | 0.062 ±0.005^a^ | 0.023 ±0.008^b^ | 0.049 ±0.012^a^ | 0.031 ±0.009^b^ | 190 | ＜0.1 | ＜0.1 | 0.156 | ＜0.1 |
| 2-Hexenoic acid, (E)- | 1957.27 | C_6_H_10_O_2_ | 13419-69-7 | ND | 0.003 ±0.002 | ND | ND |  |  |  |  |  |
| Heptanoic acid | 1943.73 | C_7_H_14_O_2_ | 111-14-8 | 0.024 ±0.004^a^ | 0.005 ±0.002^b^ | 0.010 ±0.002^b^ | 0.009 ±0.005^b^ | 640 | ＜0.1 | ＜0.1 | ＜0.1 | ＜0.1 |
| Pentanoic acid, 4-methyl- | 1793.68 | C_6_H_12_O_2_ | 646-07-1 | 0.002 ±0.001 | 0.002 ±0.001 | 0.002 ±0.001 | 0.003 ±0.001 | 810 | ＜0.1 | ＜0.1 | ＜0.1 | ＜0.1 |
| Hexanoic acid, 2-ethyl- | 1942.28 | C_8_H_16_O_2_ | 149-57-5 | 0.006 ±0 | 0.004 ±0.002 | 0.005 ±0.002 | 0.006 ±0.002 |  |  |  |  |  |
| Butanoic acid, 2-methyl- | 1660.58 | C_5_H_10_O_2_ | 116-53-0 | 0.013 ±0.003 | 0.006 ±0.002 | 0.012 ±0.01 | 0.007 ±0.002 | 10 | 0.195 | ＜0.1 | 0.706 | 0.113 |
| SUM |  |  |  | 1.183 | 0.531 | 1.221 | 1.381 |  |  |  |  |  |

Table S4 (continued)

| Compounds | RI | Formula | CAS | Content/mg/mL | | | | Threshold/ppb | ROAV | | | |
| --- | --- | --- | --- | --- | --- | --- | --- | --- | --- | --- | --- | --- |
|  |  |  |  | WO | OO | FSO | CO |  | WO | OO | FSO | CO |
| **Terpenoids** |  |  |  |  |  |  |  |  |  |  |  |  |
| D-Limonene | 1181.07 | C_10_H16 | 5989-27-5 | 0.076 ±0.004 | 0.066 ±0.064 | 0.044 ±0.01 | 0.031 ±0.006 |  |  |  |  |  |
| α-Farnesene | 1739.2 | C_15_H_24_ | 502-61-4 | ND | 0.001 ±0 | ND | ND |  |  |  |  |  |
| Bicyclo[3.1.0]hexane, 4-methylene-1-(1-methylethyl)- | 1102.36 | C_10_H_16_ | 3387-41-5 | 0.040 ±0.003^a^ | ND | 0.029 ±0.006^b^ | 0.021 ±0.005^c^ |  |  |  |  |  |
| ç-Terpinene | 1229.73 | C_10_H_16_ | 99-85-4 | 0.002 ±0 | 0.001 ±0 | ND | 0.002 ±0.001 |  |  |  |  |  |
| α-Phellandrene | 1018.24 | C_10_H_16_ | 99-83-2 | 0.008 ±0.001 | 0.014 ±0.006 | 0.010 ±0.005 | 0.007 ±0.003 | 40 | ＜0.1 | ＜0.1 | 0.145 | ＜0.1 |
| β-Myrcene | 1151.12 | C_10_H_16_ | 123-35-3 | 0.033 ±0^a^ | ND | 0.024 ±0.005^b^ | 0.015 ±0.001^c^ | 13 | 0.382 | ＜0.1 | 1.117 | 0.185 |
| 1,3,6-Octatriene, 3,7-dimethyl-, (Z)- | 1239.775 | C_10_H_16_ | 3338-55-4 | ND | 0.006 ±0.002 | ND | ND |  |  |  |  |  |
| SUM |  |  |  | 0.158 | 0.087 | 0.106 | 0.075 |  |  |  |  |  |
| **Ketones** |  |  |  |  |  |  |  |  |  |  |  |  |
| 3-Pentanone | 964.85 | C_5_H_10_O | 96-22-0 | ND | 0.464 ±0.106 | ND | ND |  |  |  |  |  |
| 2-Heptanone | 1171.2 | C_7_H_14_O | 110-43-0 | 0.021 ±0.002^c^ | 0.008 ±0.003^d^ | 0.041 ±0.001^b^ | 0.064 ±0.002^a^ | 20 | 0.162 | ＜0.1 | 1.252 | 0.523 |
| Acetophenone | 1629.22 | C_8_H_8_O | 98-86-2 | 0.003 ±0 | 0.003 ±0.001 | 0.003 ±0 | 0.003 ±0 | 65 | ＜0.1 | ＜0.1 | ＜0.1 | ＜0.1 |
| Isophorone | 1576.88 | C_9_H_14_O | 78-59-1 | 0.039 ±0.012 | 0.023 ±0.01 | 0.031 ±0.007 | 0.027 ±0.018 | 2000 | ＜0.1 | ＜0.1 | ＜0.1 | ＜0.1 |
| 2-Cyclopenten-1-one, 2,3-dimethyl- | 1430.93 | C_7_H_10_O | 1121-05-7 | ND | ND | 0.008 ±0 | ND |  |  |  |  |  |
| 2-Octanone | 1273.85 | C_8_H_16_O | 111-13-7 | 0.016 ±0.001 | 0.013 ±0.004 | 0.016 ±0.001 | ND | 41 | ＜0.1 | ＜0.1 | 0.237 | ＜0.1 |
| 3,5-Octadien-2-one, (E,E)- | 1556.27 | C_8_H_12_O | 1871-98-6 | ND | ND | 0.032 ±0.003^a^ | 0.004 ±0.001^b^ |  |  |  |  |  |

Table S4 (continued)

| Compounds | RI | Formula | CAS | Content/mg/mL | | | | Threshold/ppb | ROAV | | | |
| --- | --- | --- | --- | --- | --- | --- | --- | --- | --- | --- | --- | --- |
|  |  |  |  | WO | OO | FSO | CO |  | WO | OO | FSO | CO |
| 1-Penten-3-one | 1011.11 | C_5_H_8_O | 1629-58-9 | 0.006 ±0.001^b^ | 0.617 ±0.241^a^ | 0.113 ±0.004^b^ | ND | 1 | 0.398 | 100 | 68.017 | ＜0.1 |
| 3,5-Octadien-2-one | 1505.61 | C_8_H_12_O | 30086-02-3 | 0.001 ±0^b^ | ND | 0.076 ±0.006^a^ | 0.010 ±0.002^b^ |  |  |  |  |  |
| 6-Methyl-3,5-heptadiene-2-one | 1578.6 | C_8_H_12_O | 1604-28-0 | ND | ND | 0.007 ±0 | ND |  |  |  |  |  |
| SUM |  |  |  | 0.088 | 1.128 | 0.327 | 0.107 |  |  |  |  |  |
| **Alkanes** |  |  |  |  |  |  |  |  |  |  |  |  |
| Dodecane | 1194.36 | C_12_H_26_ | 112-40-3 | 0.022 ±0.002 | 0.016 ±0.005 | 0.022 ±0.019 | 0.021 ±0.005 |  |  |  |  |  |
| Decane, 4-methyl- | 1051.22 | C_11_H_24_ | 2847-72-5 | 0.009 ±0.001 | 0.006 ±0.002 | 0.008 ±0.003 | 0.007 ±0.001 |  |  |  |  |  |
| Pentadecane | 1497.81 | C_15_H_32_ | 629-62-9 | 0.019 ±0.002^a^ | 0.003 ±0.001^b^ | 0.013 ±0.01^ab^ | 0.015 ±0.004^a^ |  |  |  |  |  |
| Hexadecane | 1598.18 | C_16_H_34_ | 544-76-3 | 0.016 ±0.001^a^ | 0.003 ±0^b^ | 0.018 ±0.015^a^ | 0.012 ±0.003^ab^ |  |  |  |  |  |
| Tetradecane | 1397.83 | C_14_H_30_ | 629-59-4 | ND | ND | ND | 0.009 ±0.004 |  |  |  |  |  |
| Decane | 1000.9 | C_10_H_22_ | 124-18-5 | 0.004±0.001^ab^ | 0.003 ±0.001^b^ | 0.006±0.001^ab^ | 0.006 ±0.002^a^ |  |  |  |  |  |
| Cyclohexane, ethyl- | 891.24 | C_8_H_16_ | 1678-91-7 | ND | ND | ND | 0.047 ±0.003 |  |  |  |  |  |
| Heptane, 4-ethyl- | 1006.75 | C_9_H_20_ | 2216-32-2 | 0.003 ±0.001 | 0.002 ±0.001 | 0.003 ±0.001 | 0.002 ±0.001 |  |  |  |  |  |
| Decane, 5-methyl- | 1047.8 | C_11_H_24_ | 13151-35-4 | 0.004 ±0.001 | 0.003 ±0.001 | 0.006 ±0 | 0.004 ±0.001 |  |  |  |  |  |
| Heptacosane | 1698.21 | C_27_H_56_ | 593-49-7 | 0.004 ±0^ab^ | 0.001 ±0^b^ | 0.007 ±0.005^a^ | 0.007 ±0.002^ab^ |  |  |  |  |  |
| Hexane, 3,3-dimethyl- | 1292.42 | C_8_H_18_ | 563-16-6 | 0.001 ±0 | 0.001 ±0 | 0.001 ±0 | 0.001 ±0 |  |  |  |  |  |
| Dodecane, 4-methyl- | 1207.63 | C_13_H_28_ | 6117-97-1 | 0.005 ±0.001 | ND | 0.007 ±0.001 | 0.004 ±0.001 |  |  |  |  |  |
| 1,1'-Bicyclopentyl | 1075.55 | C_10_H_18_ | 1636-39-1 | 0.002 ±0.001^b^ | 0.035 ±0.031^a^ | ND | ND |  |  |  |  |  |
| Octadecane | 1245.97 | C_18_H_38_ | 593-45-3 | 0.017 ±0.003 | 0.011 ±0.004 | 0.013 ±0.012 | 0.012 ±0.001 |  |  |  |  |  |
| Octane, 3-methyl- | 949.31 | C_9_H_20_ | 2216-33-3 | 0.002 ±0.001^a^ | ND | ND | 0.001 ±0^b^ |  |  |  |  |  |
| Hexane, 2,4-dimethyl- | 1088.6 | C_8_H_18_ | 589-43-5 | 0.004 ±0.003^b^ | 0.004 ±0.001^b^ | 0.008 ±0.001^a^ | 0.005 ±0.002^ab^ |  |  |  |  |  |

Table S4 (continued)

| Compounds | RI | Formula | CAS | Content/mg/mL | | | | Threshold/ppb | ROAV | | | |
| --- | --- | --- | --- | --- | --- | --- | --- | --- | --- | --- | --- | --- |
|  |  |  |  | WO | OO | FSO | CO |  | WO | OO | FSO | CO |
| Heptane,2,2,3,3,5,6,6-heptamethyl- | 1217.04 | C_14_H_30_ | 7225-67-4 | 0.003±0.001^ab^ | 0.002 ±0.001^b^ | 0.005 ±0^a^ | 0.002 ±0.001^b^ |  |  |  |  |  |
| SUM |  |  |  | 0.114 | 0.091 | 0.117 | 0.16 |  |  |  |  |  |
| **Alkenes** |  |  |  |  |  |  |  |  |  |  |  |  |
| 1-Pentadecene | 1236.9 | C_15_H_30_ | 13360-61-7 | 0.001 ±0^b^ | 0.014 ±0.004^a^ | 0.001 ±0^b^ | ND |  |  |  |  |  |
| 3-Hexene, 2,2-dimethyl-, (Z)- | 1869.67 | C_8_H_16_ | 690-92-6 | ND | ND | 0.004 ±0 | ND |  |  |  |  |  |
| Decane, 4-methylene- | 1766.7 | C_11_H_22_ | 24949-41-5 | ND | ND | 0.004 ±0 | ND |  |  |  |  |  |
| SUM |  |  |  | 0.001 | 0.014 | 0.009 | 0 |  |  |  |  |  |
| **Organosulfur compounds** | | | | | | | | | | | | |
| Di-tert-butyl disulfide | 1300.3 | C_8_H_18_S_2_ | 110-06-5 | ND | ND | 0.038±0.014 | ND |  |  |  |  |  |
| 1-Butene, 4-isothiocyanato- | 1438.26 | C_5_H_7_NS | 3386-97-8 | 0.448 ±0.036^a^ | ND | 0.023 ±0.019^b^ | 0.002 ±0.001^b^ |  |  |  |  |  |
| Benzoyl isothiocyanate | 1897.48 | C_8_H_5_NOS | 532-55-8 | 0.003 ±0 | 0.004 ±0.003 | 0.003 ±0.002 | 0.005 ±0.002 |  |  |  |  |  |
| SUM |  |  |  | 0.451 | 0.004 | 0.064 | 0.007 |  |  |  |  |  |
| **Heterocyclic compounds (pyridines)** | | | | | | | | | | | | |
| Pyridine | 1172.38 | C_5_H_5_N | 110-86-1 | 0.148 ±0.013^a^ | 0.063 ±0.059^b^ | 0.062 ±0.042^b^ | 0.081 ±0.008^ab^ | 820 | ＜0.1 | ＜0.1 | ＜0.1 | ＜0.1 |
| SUM |  |  |  | 0.148 | 0.063 | 0.062 | 0.081 |  |  |  |  |  |
| **Heterocyclic compounds (furans)** | | | | | | | | | | | | |
| Furan, 2-pentyl- | 1218.84 | C_9_H_14_O | 3777-69-3 | 0.037 ±0.003^b^ | ND | 0.095 ±0.006^a^ | 0.005 ±0.001^c^ | 6 | 0.927 | ＜0.1 | 9.538 | 0.144 |
| Furan, 2-ethyl- | 939.01 | C_6_H_8_O | 3208-16-0 | 0.006 ±0.001^b^ | ND | 0.284 ±0.007^a^ | 0.003 ±0.001^b^ |  |  |  |  |  |
| Furan, 2-methyl- | 893.98 | C_5_H_6_O | 534-22-5 | ND | ND | 0.020 ±0.001 | ND | 3500 | ＜0.1 | ＜0.1 | ＜0.1 | ＜0.1 |

Table S4 (continued)

| Compounds | RI | Formula | CAS | Content/mg/mL | | | | Threshold/ppb | ROAV | | | |
| --- | --- | --- | --- | --- | --- | --- | --- | --- | --- | --- | --- | --- |
|  |  |  |  | WO | OO | FSO | CO |  | WO | OO | FSO | CO |
| Furan, 2,3-dihydro-4-methyl- | 1184.03 | C_5_H_8_O | 34314-83-5 | ND | ND | 0.017 ±0.001 | ND |  |  |  |  |  |
| SUM |  |  |  | 0.043 | 0 | 0.416 | 0.008 |  |  |  |  |  |
| **Heterocyclic compounds (furanones)** | | | | | | | | | | | | |
| 2(3H)-Furanone, 5-butyldihydro- | 1679.98 | C_6_H_10_O_2_ | 695-06-7 | 0.044 ±0.003^b^ | 0.001 ±0^c^ | 0.108 ±0.004^a^ | 0.006 ±0.002^c^ | 1600 | ＜0.1 | ＜0.1 | ＜0.1 | ＜0.1 |
| 2(3H)-Furanone, 5-butyldihydro- | 1896.57 | C_8_H_14_O_2_ | 104-50-7 | 0.004 ±0^b^ | ND | 0.006 ±0.001^a^ | ND |  |  |  |  |  |
| SUM |  |  |  | 0.048 | 0.001 | 0.113 | 0.006 |  |  |  |  |  |
| **Heterocyclic compounds (thiazoles)** | | | | | | | | | | | | |
| 2-Acetylthiazole | 1626.8 | C_5_H_5_NOS | 24295-03-2 | ND | 0.001 ±0 | ND | ND | 4 | ＜0.1 | ＜0.1 | ＜0.1 | ＜0.1 |
| SUM |  |  |  | 0 | 0.001 | 0 | 0 |  |  |  |  |  |
| **Esters** |  |  |  |  |  |  |  |  |  |  |  |  |
| sec-Butyl acetate | 978.09 | C_6_H_12_O_2_ | 105-46-4 | 0.133 ±0.004 | ND | ND | ND |  |  |  |  |  |
| Pentanoic acid, 3-methyl-, methyl ester | 1119.9 | C_7_H_14_O_2_ | 2177-78-8 | 0.108 ±0.005 | ND | ND | ND |  |  |  |  |  |
| Pentanedioic acid, dimethyl ester | 1686.1 | C_7_H_12_O_4_ | 1119-40-0 | 0.004 ±0^ab^ | 0.003 ±0.001^b^ | 0.005 ±0.001^a^ | 0.004 ±0.001^ab^ |  |  |  |  |  |
| Butyrolactone | 1601.9 | C_4_H_6_O_2_ | 96-48-0 | 0.013 ±0.001^b^ | 0.002 ±0.001^c^ | 0.029 ±0.001^a^ | 0.003 ±0.001^c^ | 20000 | ＜0.1 | ＜0.1 | ＜0.1 | ＜0.1 |
| Hexanoic acid, ethyl ester | 1225.11 | C_8_H_16_O_2_ | 123-66-0 | 0.009 ±0^ab^ | 0.006 ±0.002^b^ | 0.011 ±0.001^a^ | 0.009 ±0.003^ab^ | 0.3 | 4.425 | 3.503 | 21.631 | 4.688 |
| Hexanedioic acid, dimethyl ester | 1805.28 | C_8_H_14_O_4_ | 627-93-0 | 0.001 ±0 | 0.001 ±0 | 0.002 ±0 | 0.001 ±0 |  |  |  |  |  |

Table S4 (continued)

| Compounds | RI | Formula | CAS | Content/mg/mL | | | | Threshold/ppb | ROAV | | | |
| --- | --- | --- | --- | --- | --- | --- | --- | --- | --- | --- | --- | --- |
|  |  |  |  | WO | OO | FSO | CO |  | WO | OO | FSO | CO |
| Linalyl acetate | 1547.51 | C_12_H_20_O_2_ | 115-95-7 | 0.006 ±0^b^ | 0.012 ±0.003^a^ | 0.001 ±0^c^ | 0.001 ±0^c^ | 1000 | ＜0.1 | ＜0.1 | ＜0.1 | ＜0.1 |
| Methyl salicylate | 1752.97 | C_8_H_8_O_3_ | 119-36-8 | ND | ND | ND | ND | 40 | ＜0.1 | ＜0.1 | ＜0.1 | ＜0.1 |
| 1-Butanol, 3-methyl-, acetate | 1113.03 | C_7_H_14_O_2_ | 123-92-2 | 0.001 ±0 | ND | ND | ND | 2 | ＜0.1 | ＜0.1 | ＜0.1 | ＜0.1 |
| 2H-Pyran-2-one, tetrahydro-6-methyl- | 1584.73 | C_6_H_10_O_2_ | 823-22-3 | 0.026 ±0.002 | ND | ND | ND |  |  |  |  |  |
| Propanoic acid, 2-hydroxy-, ethyl ester, (L)- | 1331.8 |  |  | 0.016 ±0.011^a^ | 0.006 ±0.005^b^ | ND | ND |  |  |  |  |  |
| SUM |  |  |  | 0.316 | 0.03 | 0.048 | 0.018 |  |  |  |  |  |
